# Loss of immune cell identity with age inferred from large atlases of single cell transcriptomes

**DOI:** 10.1111/acel.14306

**Published:** 2024-08-14

**Authors:** Erin Connolly, Tony Pan, Maneesha Aluru, Sriram Chockalingam, Vishal Dhere, Greg Gibson

**Affiliations:** ^1^ Center for Integrative Genomics, and School of Biological Sciences Georgia Institute of Technology Atlanta Georgia USA; ^2^ Institute for Data Science and Engineering, Georgia Institute of Technology Atlanta Georgia USA; ^3^ Department of Biomedical Informatics Emory University Atlanta Georgia USA; ^4^ Winship Cancer Institute, Department of Radiation Oncology Emory University Hospital Midtown Atlanta Georgia USA

**Keywords:** computational pipeline, differential expression, immune cell aging, peripheral blood mononuclear cells, scRNAseq

## Abstract

By analyzing two large atlases of almost 4 million cells, we show that immune‐senescence involves a gradual loss of cellular identity, reflecting increased cellular heterogeneity, for effector, and cytotoxic immune cells. The effects are largely similar in both males and females and were robustly reproduced in two atlases, one assembled from 35 diverse studies including 678 adults, the other the OneK1K study of 982 adults. Since the mean transcriptional differences among cell‐types remain constant across age deciles, there is little evidence for the alternative mechanism of convergence of cell‐type identity. Key pathways promoting activation and stemness are down‐regulated in aged T cells, while CD8 TEM and CD4 CTLs exhibited elevated inflammatory, and cytotoxicity in older individuals. Elevated inflammatory signaling pathways, such as MAPK and TNF‐alpha signaling via NF‐kB, also occur across all aged immune cells, particularly amongst effector immune cells. This finding of lost transcriptional identity with age carries several implications, spanning from a fundamental biological understanding of aging mechanisms to clinical perspectives on the efficacy of immunomodulation in elderly people.

## Graphical abstract

We utilize two large atlases of single cell PBMC expression to illustrate decline in marker identity with age in humans across multiple cell types. This is largely due to increasing variability among individuals rather than convergence of identity, and involved inflammatory, stemness, and cytotoxicity pathways.

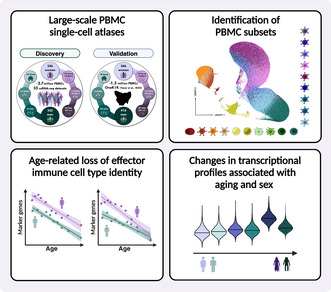

**Abbreviations**
BC: B cell
CTL: cytotoxic T cell
DC: dendritic cell
DE: differentially expressed
DEG: differentially expressed gene
ENA: European nucleotide archive
GEO: gene expression omnibus
IFN: interferon
LFC: log fold change
LR: linear regression
MAIT: mucosal‐associated invariant T cells
MAPK: mitogen‐activated protein kinase
MC: monocyte
MHC: major histocompatibility complexes
mRNA: messenger RNA
mTORC1: mammalian target of rapamycin complex 1
NF‐kB: Nuclear factor kappa‐light‐chain‐enhancer of activated B cells
NK: natural killer cell
OF: older female
OM: older male
Oxphos: oxidative phosphorylation
PBMC: peripheral blood mononuclear cells
PCA: principal component analysis
SCP: Single cell portal
scRNA‐seq: single‐cell transcriptomic sequencing
SLE: Systemic Lupus Erythematosus
TCM: Central memory T cell
TEM: Effector memory T cell
TNF: Tumor necrosis factor
Tregs: Regulatory T cells
UMAP: uniform manifold approximation and projection
UMI: unique molecular identifiers
XIST: X‐inactive specific transcript
YF: young female
YM: young male

## 1 INTRODUCTION

Aging of the human immune system is characterized broadly by relative loss of naïve lymphocytes, and gain of cytotoxic T‐cells as well as plasma cells (Nikolich‐Žugich, 2018; Sadighi Akha, 2018). Sex differences in the rate and scope of these changes likely contribute to divergent functional immunological responses including susceptibility to autoimmune disease in females and lethal malignancy and inflammatory disease in males (Huang et al., 2021; Márquez et al., 2020). Single cell transcriptomics is beginning to supplement traditional flow cytometry (Cole et al., 2021; Luo et al., 2022; Zhu et al., 2023), but to date sample sizes have been too small to account for diverse covariates that may contribute to heterogeneous peripheral blood profiles.

While it has long been appreciated that one of the hallmarks of cellular aging is immune‐senescence (Boraschi et al., 2010; Liu et al., 2023; Panda et al., 2009), currently there are no high‐resolution single cell data available evaluating whether immune‐senescence also involves gradual loss of immune cellular identity. Recently published work by Izgi et al. (2022) and the work of others (Cardoso‐Moreira et al., 2019) indicates that mammalian tissue transcriptome profiles diverge from one another other during development but are less distinct with aging. One potential hypothesis to explain this phenomenon is that with aging, tissues gradually shift towards decreased specialization resulting in increased heterogeneity and transcriptomic overlap. Such a late‐age phenomenon would be compatible with the idea of aging‐related cellular identity loss. Additionally, documenting the loss of immune cellular identity across sexes is critical to better understand the prevalence, mechanisms, and functional consequences in human aging. However, progress has been constrained by the limited availability of studies incorporating both substantial sample sizes and encompassing the entire human lifespan across diverse immune cell types.

This study offers a temporal analysis of age‐related changes of single‐cell transcriptome profiles of humans, including samples from 19 of the most prevalent PBMC immune cell types across ten aging periods that span the entire postnatal lifespan. In addition to confirming established alterations in cell type abundance, we evaluate the hypothesis that one of the hallmarks of cellular aging is loss of cellular identity (Cardoso‐Moreira et al., 2019; Izgi et al., 2022), most notably in effector cell populations. Utilizing a novel encoding that speeds up the Wilcoxon Rank Sum test, which is commonly used in Seurat to find differentially expressed (DE) marker genes for each cluster of cells (Butler et al., 2018; Satija et al., 2015), we quantify linear alterations of the number of markers per cell type stratified by age decile. These measures of cellular identity replicate across both sexes and are remarkably consistent in a validation atlas of ~1.2 million PBMC single cell RNAseq profiles from ~1200 cells for each of 982 adults in the Australian OneK1K study (Yazar et al., 2022). Furthermore, these findings of increasing cellular entropy with age are confirmed by linear mixed modeling of pseudobulk profiles, and we provide evidence that the effect is due to increased heterogeneity within aged cell types rather than loss of identity divergence between cell types.

## 2 RESULTS

### 2.1 Study design and analysis of single immune cell profiling by sex and aging with FastDE

**FIGURE 1:**
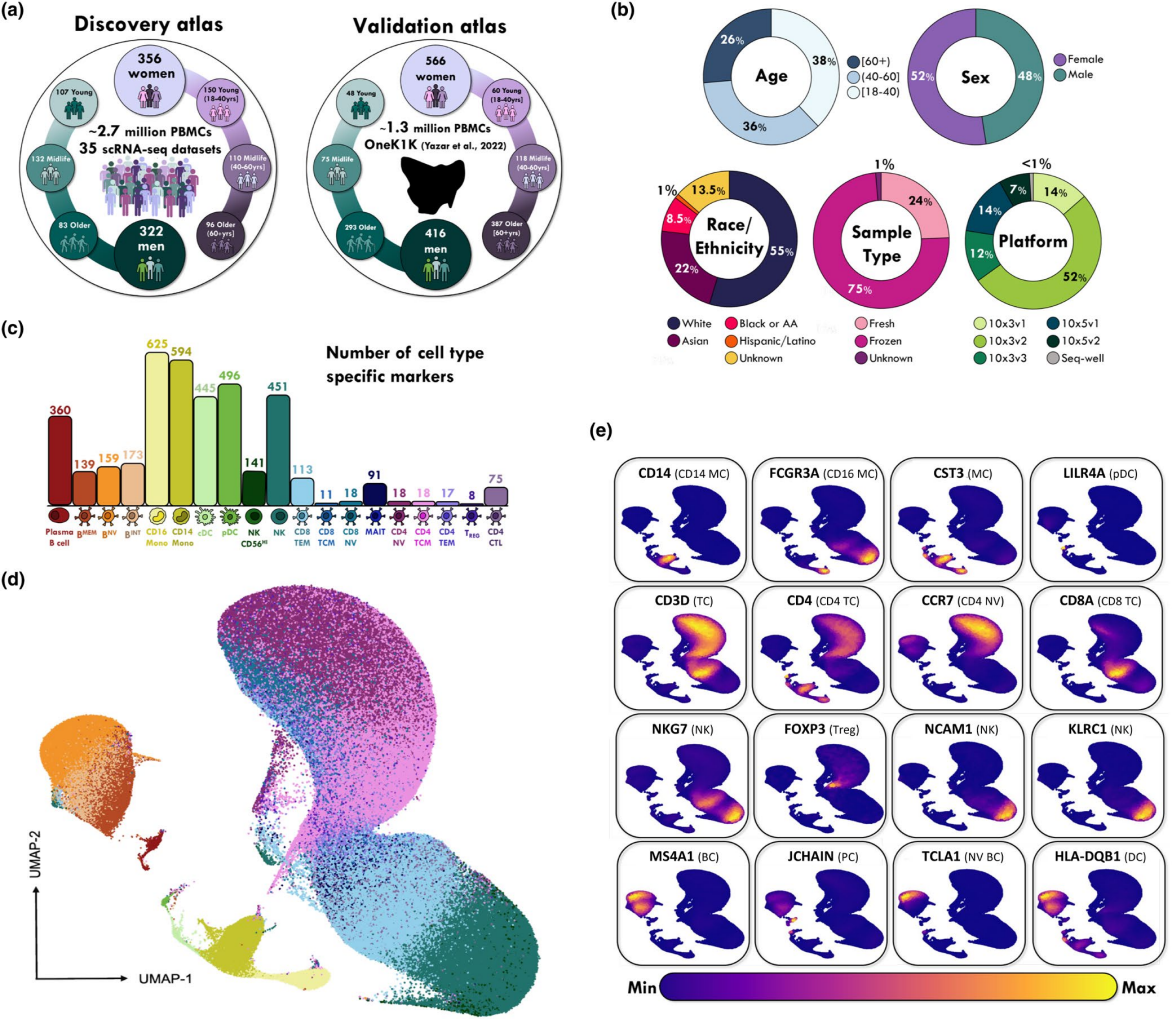
Overview of the approach and clustering strategy used for analyzing the circulating immune cell profiling of aging and sex. (a) scRNA‐seq data of PBMCs from a discovery cohort comprising of 356 women and 322 men (18–110 years old) and the independent 1.2 millioncell OneK1K validation atlas from 982 Australian (Tasmanian) adults (18–97 years old). (b) Donor demographics and sample type displayed as color‐coded donut plots. (c) Bar plot showing the number of marker genes for each of 19 cell types from all 2.74 M cells in the discovery atlas.(d) UMAP of PBMCs across all individuals in the discovery atlas, with 19 transcriptionally distinct populations. (e) Density plots of canonical markers of immune cells in discovery atlas. Density corresponding to expression of the labelled gene. Cells are colored based on the expression of the transcript, bottom legend.

**FIGURE 2:**
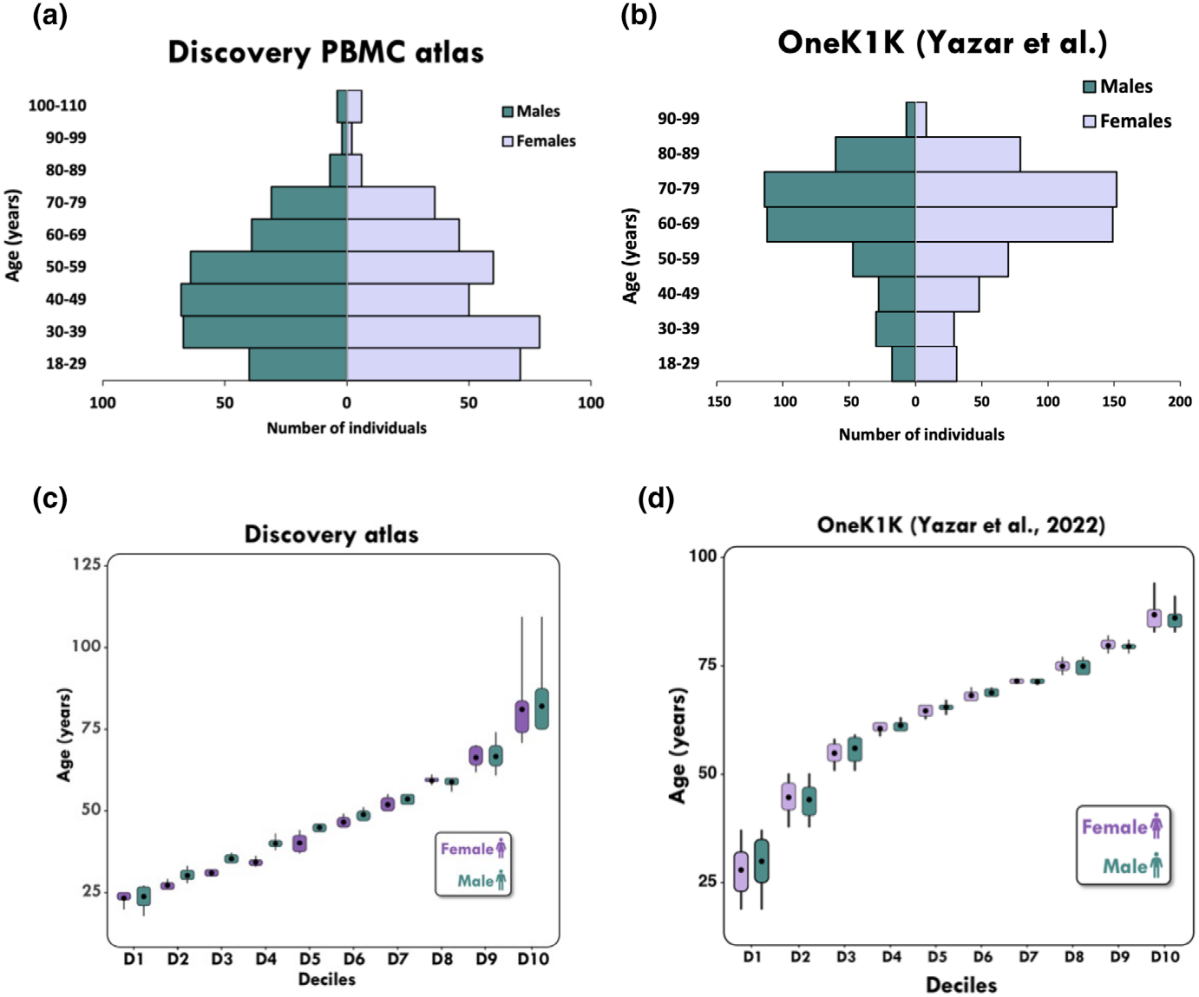
Distribution of age and sex in the (a) discovery and (b) OneK1K cohorts. (c–d) Age distribution for deciles of the PBMC data atlases. Healthy subjects were divided into 20 groups (10 age deciles for males and 10 age deciles for females) according to sex and the age distributions for Discovery Atlas and OneK1K.

We assembled an atlas of ~2.7 million PBMC single cell RNAseq profiles from 35 studies, collectively sampling 678 adults across the lifespan (Figure 1a). For each contributing study listed in Supplemental Material Table S1, PBMC clusters were identified and imported into a common Seurat object, focusing on 19 common cell types robustly identified in all contributing datasets: naïve, intermediate and memory B cells, plasma cells, conventional and plasmacytoid dendritic cells, CD14 and CD16 monocytes, Cytotoxic CD4 T cells, NK cells, CD56+ NK cells, MAIT cells, central and effector memory and naive CD4+ and CD8+ T cells, and regulatory T cells (Tregs) (Figure 1c,d). We partitioned the dataset into male and female subsets and equilibrated the number of cells in each age decile and sex (Figure 2a,c) to facilitate equivalent statistical power for marker detection. As described in the Methods, preliminary analyses showed that partitioning by age class results in imbalances that strongly influence performance of the Wilcoxon test for differential expression (Figure S1).

Single cell profiling is generally subject to batch effects (Tran et al., 2020) where heterogeneity can arise not only from sample‐to‐sample technical issues, but also biological differences. To address these potential limitations, we confirmed that batch correction was successful, and that all included datasets were merged homogeneously by using the transfer‐learning method Azimuth (Hao et al., 2021), which mapped cell‐type annotations from published PBMC datasets onto the atlas. The cell‐type assignments were confirmed on the basis of transcriptomic similarity, which showed high cell type concordance pre and post integration (Figure S2). The overall cell‐type composition of the discovery atlas is shown in Figure 1b, and the composition of each cohort in terms of (i) age range, (ii) sex, (iii) donor ethnicity, and (iv) median reads per cell are provided in Table S1.

Since Seurat took 40 min to process a 600 K cell dataset on a 64‐core processor (almost 2 days on a single core) and was unable to handle the full atlas, we rewrote aspects of the code to facilitate differential expression analysis. Specifically, the FindMarkers function consumed between 79% and 97% of the runtime, and was slowed by use of a dense rather than sparse matrix as well as unnecessary repetition of calculations. As described in the Methods, our FastDE implementation of the Mann–Whitney‐*U* (Wilcoxon Rank‐Sum) test, available open source at CRAN or GitHub and coded in C++ (Stroustrup, 2013), follows the approach of BioQC (Zhang et al., 2017) of sorting the observed counts for each gene once and reusing the sorted rank for comparing each cluster against all other clusters. Memory usage is also optimized, by deploying sequential access to a small cache‐friendly hash table, generating tie correction factors during summation, and assigning zero counts the same rank. These changes facilitate computation on the 2.7 M cell dataset in 45 s on 64 cores, with comparable speed‐up with subsets of the dataset.

FastDE was also able to support processing of the entire atlas, and additionally demonstrated that young (18–40 years old) and middle‐aged (41–60 years old) adult cell‐types closely resemble the ensemble profile, thus providing a reasonable approximation of the marker identities amongst these 19 predominant immune cell types in human blood. Complete lists of markers are provided in Table S2.

### 2.2 Changes in cell proportions associated with aging and sex

We first confirmed previous observations of change in cell proportions with age and sex. Figure S3 illustrates that there is a gradual replacement of naïve CD4+ and CD8+ T‐cells by central and effector memory T‐cells respectively, as well as an expansion of the NK cell population, in each case almost doubling the proportions, matching observations reported elsewhere (Huang et al., 2021). CD14+ and CD16+ monocytes also gradually increase in proportion with age. The total B cell population remains constant with no clear patterns of gain or loss of naïve and memory B cells or plasma cells. Interestingly, a difference in cell type proportion between sexes is seen most prominently in younger individuals. These sex differences are retained with age in monocytes, NK cells, and CD4 T cells, however, are lost in CD8 T cells.

### 2.3 Loss of effector immune cell identity with age

**FIGURE 3:**
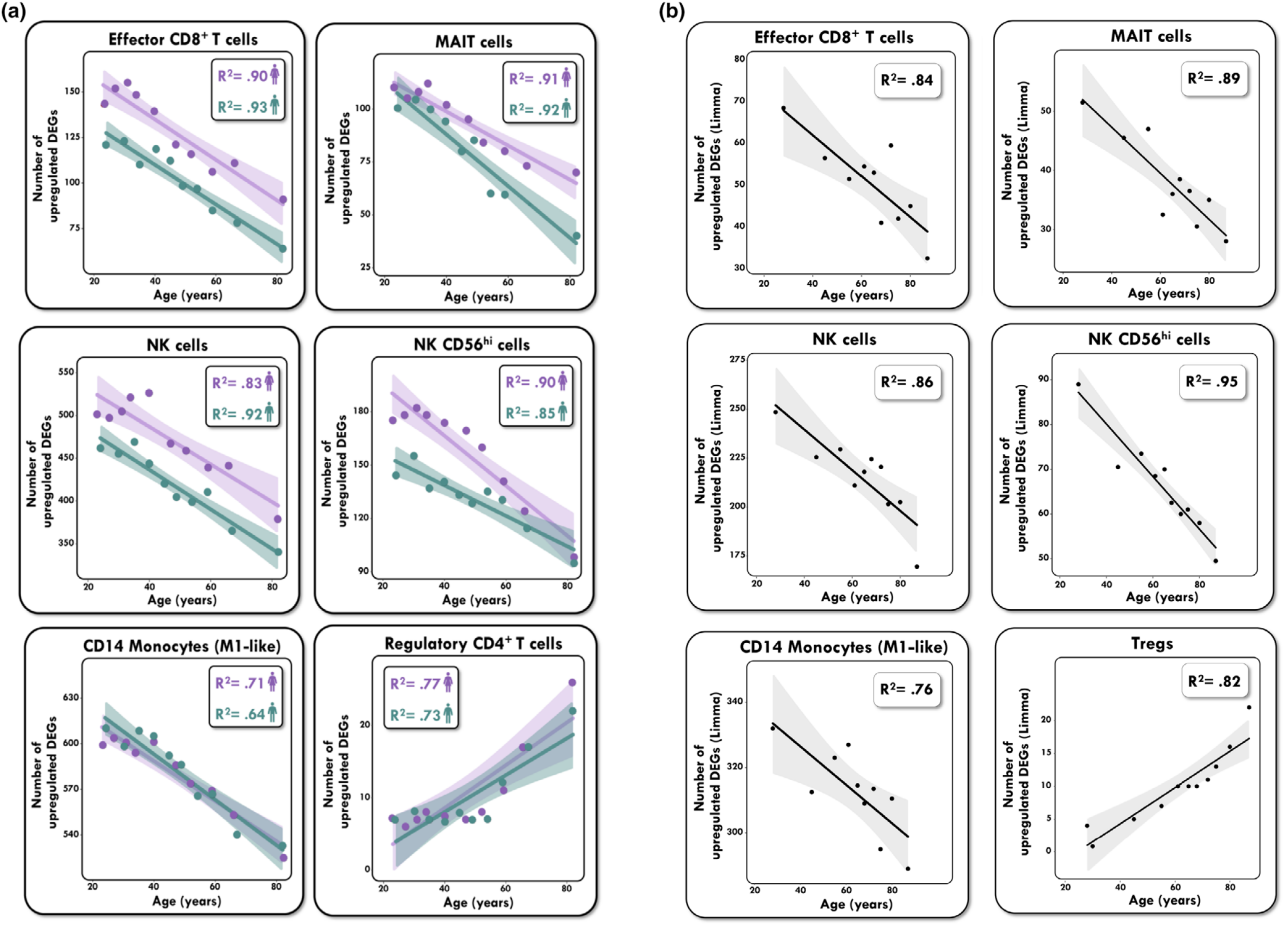
Loss of effector immune cell identity with age (a) Positive correlation between age and the number of cell‐type specific markers in effector immune cells (CD8 effector T cells; MAIT cells; NK cells; NK CD56+ cells; CD14 Monocytes). Negative correlation between age and the number of cell type specific markers in CD4 regulatory T cells. DEG analysis performed with FastDE (b) DEG analysis replicated with limma. See Figure S4 for full set of cell types.

Results reported in Figure 3 and Figure S4 support the inference that effector and cytotoxic immune cell types are less clearly differentiated as people age. Notably, the opposite phenomenon was true in Treg and Naïve CD4 T cells (Figure 3a,b), with both cell types undergoing clear gain of cell‐type identity markers in elderly males and females. These findings are replicated in an independent atlas of 1.2 million cells from almost 1000 healthy donors in the OneK1K study (Yazar et al., 2022). Despite observing a slightly older age pyramid in OneK1K (Figure 2b), the core findings that effector immune cell types have less divergent gene expression profiles while Tregs and Naïve CD4 T cells undergo an age‐related gain in cell‐type identity in the elderly population, over the age of 60, replicated with no exceptions (Figure S5). Note that the *y*‐axes in these figures are plotted on cell type‐ specific scales.

While our analyses include extensive biological replicates (individual donors), the Wilcoxon rank‐sum method used in FastDE treats each cell as its own biological replicate, which can lead to an inflation of estimates of statistical significance due to the large number of comparisons made. To address this issue and further assess the reproducibility of our findings, we adopted a pseudobulk workflow using the Dreamlet tool (Hoffman et al., 2023) to aggregate expression values from all cells assigned to each cell type within each subject. This provides a more robust and reliable estimation of differential gene expression among individuals while mitigating the effects of noise and study‐specific variability at the single‐cell level (though it precludes evaluation of within cell‐type variability within individuals). The *snm* package (Mecham et al., 2010) was applied to the pseudobulked data to model and remove the effects of adjustment variables including sex and sample pool without influencing the biological variable of age. Variance partitioning analysis of OneK1K for each gene across PBMC cell types was then used to estimate the fraction of expression variance attributable to variation across technical pools, age, and sex (Figure S6). Although the median fraction of variation explained by technical pools ranges from 25%–65%, more than 10% of expression variance was attributed to age across immune cell types. DEG analysis performed with the limma package (Ritchie et al., 2015) then supported the inference that effector and cytotoxic immune cell types are less clearly differentiated as people age (Figure 3b), comprehensively replicating results reported using FastDE. Furthermore, to provide a visual sense of the range of inter‐individual variability in the loss of gene expression, we also provide regression plots of the number of DEG against age for representative cell types in Figure S7. The r‐squared values range from 0.43 to 0.69 providing further validation that binning age deciles reasonable represents the age‐dependence of gene expression while emphasizing that profiles for individual donors may diverge at different rates.

### 2.4 Age‐dependent accumulation of transcriptome heterogeneity in immune cells

**FIGURE 4:**
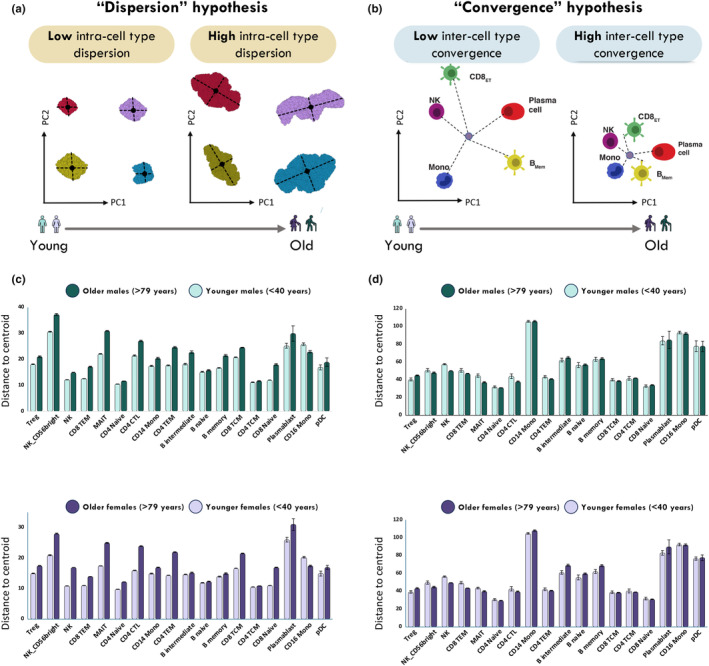
Two alternative hypotheses on the origin of age‐related loss of cell type identity. (a) The “dispersion” hypothesis postulates that the mean intrinsic dispersion for within each cell‐type increases with aging. (b) The “convergence” hypothesis proposes that immune cell types are extrinsically heterogenous in younger individuals. However, with aging, heterogeneity is lost, and cell types converge due to the reduced repertoire of cell type‐specific genes expressed. (c) Bar plot depicting the mean and standard error for the Euclidean distance from each cell to the centroid within the subset in young (<40 years) and older (79+ years) males and females. (d) Bar plot depicting the mean and standard error for the Euclidean distance from each cell to the centroid between all subsets in young (<40 years) and older (79+ years) males and females.

The most parsimonious interpretation of this result is that increased heterogeneity of profiles within cell types with age results in fewer genes being observed to be DE among cell types (Figure 4a). Such a result was recently observed for cytotoxic CD4+ T‐cells in supercentenarians (Hashimoto et al., 2019), which induce expression of some CD8+ T‐cells and gain the capacity for secretion of IFNγ and TNFα upon stimulation. However, an alternative explanation would be that greater convergence of gene expression within cells of each cluster obscures mean differences (Figure 4b). To test whether cell heterogeneity and transcriptome variability were affected upon aging, and to investigate if this could explain observed phenotypic changes such as loss of cell type identity and effector bias, we applied Disscat across PBMC immune cell types from young (<40 years old) and aged (>79 years old) individuals. Disscat was used to compute the Euclidian distance between each cell in a cluster and the centroid of that cluster, and then to compare these differences between age classes. We avoided potential confounding of noise quantification caused by batch integration by focusing our analysis on the OneK1K atlas. Within each cell type, we observed evaluated gene expression variances measured as overdispersion in aged individuals for most of the 19 cell types (Figure 4c). These findings, which are highly concordant between males and females, suggest that immune cells in the bloodstream may exhibit increased diversity and a broader range of gene expression, potentially associated with accumulating exposure to extracellular antigens during aging (Martinez‐Jimenez et al., 2017). In contrast, there was minimal change in the deviation between the distance of each cell within a cluster and the overall centroid between young and elderly samples based on the first two principal components of gene expression (Figure 4d), arguing against the convergence hypothesis.

### 2.5 Shared and sex‐specific transcriptional signatures of aging

**FIGURE 5:**
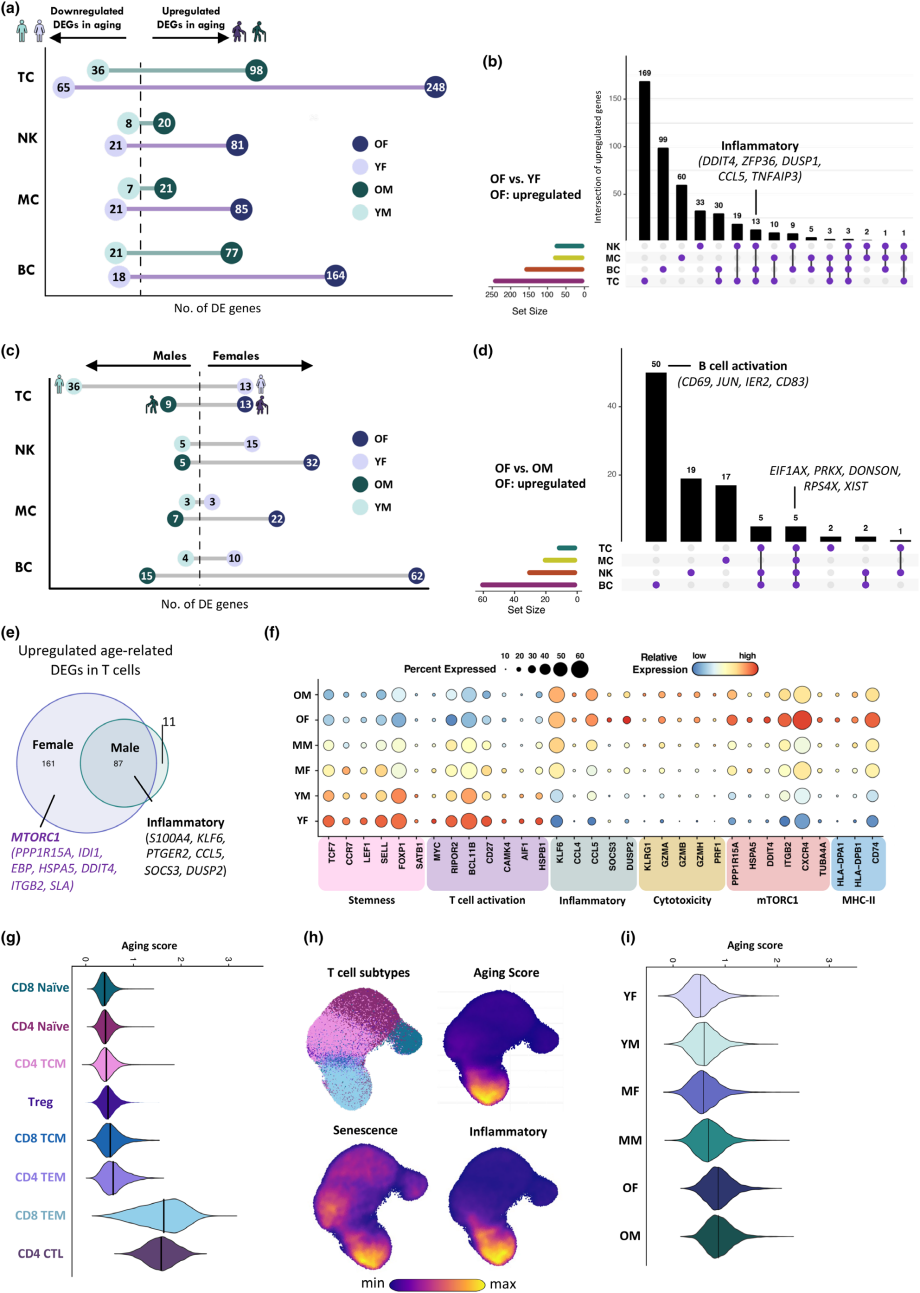
Changes in transcriptional profiles during aging. (a) Numbers of age‐related differentially expressed genes (DEGs) in four immune cell subsets comparing older males (OM) to younger males (YM) and older females (OF) to younger females (YF). (b) UpSet Plot showing the integrated comparative analysis of upregulated DEGs in major immune cell lineages between OF and YF Upregulated DEGs: Upregulated in OF, downregulated in YF. (c) Numbers of sex‐related DEGs.(YM: YF and OM: OF) in four immune cell subsets. (d) UpSet Plot showing the integrated comparative analysis of upregulated DEGs in four major immune cell lineages between OF and OM. Upregulated DEGs: Upregulated in OF, downregulated in OM. (e) Venn diagram showing integrated comparative analysis of upregulated age‐related DEGs in T cells between females and males. (f) Representative GO terms and pathways enriched in age‐related DEGs based on functional enrichment analysis in T cells of females and males. (g) Distribution and comparison of the aging score in T cell subpopulations. (h) Density plots of aging score and selected gene modules in T cells. (i) Distribution and comparison of the aging score in all T cells of each age group. (CD4 TCM, CD4+ Central Memory T cells; CD8 TCM, CD8+ Central Memory T cells; CD4 TEM, CD4+ Effector Memory T cells; CD8 TEM, CD8+ Effector Memory T cells; CD4 CTL, CD4+ Cytotoxic T cells).

To identify cell‐subtype‐specific gene signatures associated with age‐related differences, we performed a sex‐stratified comparative analysis of DEGs between older males (OM) and younger males (YM), as well as between older females (OF) and younger females (YF), within four major broadly defined cell types. We identified DEGs meeting significance and log fold change (LFC) thresholds in all cell types except DCs between age groups when stratified by sex. A complete lists of DEGs is provided in Table S3. We found that blood immune cells showed heterogeneous transcriptional changes affected by aging based on the number of DEGs in both females and males, however, the number of DEGs was substantially higher in females (Figure 5a). Strikingly, T cells (TC) were the cell types most affected by aging in both sexes, followed by B cells (BC) and then NK cells (NK) and monocytes (MC). Notably, in females, we found a set of 13 aging‐related genes whose expression was increased in TC, BC, and NK (Figure 5b). These 13 genes were within pathways commonly associated with inflammation, including MAPK signaling pathways (e.g., *MAP4K8*, *PTPN7*, *AREG*) and TNF‐alpha signaling via NF‐kB (e.g., *ZFP36*, *DUSP1*, *CCL5*, *TNFAIP3*).

To partition the contributions of sex to cell type variance in aging, we compared the DEGs between sexes (YM: YF and OM: OF) in each subset. Sex differences were evident across a broad range of immune cells and for BC, MC, and NK, increased with age (Figure 5c). Interestingly, DEG analysis revealed that more sex‐related DEGs were present in T cells at a young age, while sex differences in B cells were most apparent in older individuals. By comparing the up‐regulated DEGs of OF to OM, we found a significant gain in gene expression for *JUN, IER2*, *CD69* and *CD83* (Figure 5d), suggestive of an increased B cell activation state in OF compared to OM. Additionally, compared with OF, OM showed increased expression of the surface marker and cell adhesion molecule *CD99* transcripts across all immune cells, which is consistent with previous studies in whole blood (Lefèvre et al., 2012). Sexual dimorphism for age‐related alterations in gene expression were also evident in the heightened expression of genes related to T cell activation in YF, and genes associated with antigen presentation and mTORC1 signaling in OF, whereas these changes were not significantly observed in males (Figure 5e,f).

DE genes associated with aging nevertheless overlapped considerably between sexes (Table S3). For example, by comparing TC DEGs in aging individuals, we identified 29 downregulated and 87 upregulated genes that were shared in OF and OM (Figure 5e). Downregulated DEGS included important molecules associated with T cell stemness (e.g., *TCF7*, *LEF1*, *CCR7*, *FOXP1*) while upregulated DEGS were attributed to inflammatory pathways (eg., *S100A4*, *KLF6*, *PTGER2*, *CCL5*, *SOCS3*, *DUSP2*) (Figure 5e). To further assess the impact of aging on circulating T cells, we selected the top 30 genes of the 116 total genes that were DE and shared between sexes in the T cell compartment, and calculated aging scores across all T cell subtypes. We found that CD8 TEM and CD4 CTLs had the highest scores (Figure 5g), suggesting an enhanced senescent and inflammatory signature in these cell populations (Figure 5h). Furthermore, our findings indicate that individuals in the elderly group consistently exhibited higher scores compared to those in the young adult group (Figure 5i), underscoring the suitability of aging‐score assessments for investigating age‐related immune dysfunction.

Together, these data uncovered a transcriptional signature of aging shared between sexes, which include gains in gene expression for pro‐inflammatory processes and cytotoxicity, and losses in expression for stemness in T cells. Interestingly, these changes were more pronounced in women, despite cohorts being comparable for age and ethnicity. Furthermore, we discovered that T cells age differently between sexes, where a significant increase in antigen presentation and mTORC1 signaling and losses in expression for genes associated with T cell activation were detected only in women.

## 3 DISCUSSION

The primary aim of this study was to explore the hypothesis that immune‐senescence involves a gradual loss of cellular identity. We found that consistent loss of marker gene expression was observed for all cytotoxic and effector cell sub‐types, despite their expansion with age. The effects are largely similar in both males and females and were robustly reproduced in a second atlas comprising 1.2 million PBMCs from the OneK1K study. We also found an age‐dependent accumulation of transcriptome heterogeneity and variability upon aging, obscuring mean differences. Finally, we showed that key pathways promoting activation and stemness are down‐regulated in aged T cells, while CD8 TEM and CD4 CTLs exhibited elevated inflammatory, and cytotoxicity in older individuals. Finally, we demonstrated that key pathways promoting activation and stemness are down‐regulated in aged T cells, while elevated inflammatory signaling pathways, such as MAPK and TNF‐alpha signaling via NF‐kB, occur across all aged immune cells. Based on these observations, we propose that aging may result in an increase in the proportion of cells exhibiting a loss of transcriptional identity, particularly amongst effector immune cells. This finding carries several implications, spanning from a fundamental biological standpoint to a clinical perspective.

Biologically, the finding that aging induces effector loss of cellular identity, possibly exacerbated by a reduction in their self‐renewal capacity is provocative. Loss of specialization has been previously shown in bulk tissue samples from the cortex, liver, lung, and muscle (Izgi et al., 2022). The immune system is known to be affected by aging, with a gradual functional decline of immune cells and an emerging effector immune bias in the blood (Mogilenko et al., 2022). It is still unclear to what extent the age‐dependent decline of the immune system can be attributed to an emerging immune heterogeneity, resulting from age‐related loss of cell‐intrinsic properties. The majority of prior work has focused on aging's impact at the tissue level rather than on the single‐cell level. However, recent studies have begun to utilize scRNA‐seq to explore aging at the single‐cell level (Luo et al., 2022; Sayed et al., 2021) though they for the most part pool male and female samples for analysis which precludes consideration of potential sex‐specific effects that we address directly. Our findings suggest increased transcriptome variability in immune cells of aged individuals. Hence, we demonstrate that single‐cell resolution analysis of cell–cell heterogeneity can yield fundamentally new biological insights.

An important aspect of immune aging is the differential decline rates among various cell types, which may be influenced by cell type‐specific half‐lives. Previous studies in mouse cells (Yang et al., 2024) and in human cells (Saha et al., 2017) have highlighted that the turnover time of cells plays a crucial role in determining whether DEGs diverge with age. Our findings support this notion, as cells that decline the fastest with age, such as CD8+ and MAIT cells, are also among the longest‐lived. This suggests that the age of a cell may not necessarily correlate with the chronological age of the individual, but rather with the cell's turnover rate. For instance, some B cells have a lifespan of decades and reflect the chronological age of the donor, while monocytes, which are constantly renewed and only live for a few days, maintain a consistent age regardless of the donor's age. This underscores that the observed changes in cell distribution and function with age are partly due to the varying lifespans of different cell types, highlighting the need to consider cell‐specific turnover times in studies of immune aging.

Clinically, there are also broad and far‐reaching implications from this work. For example, a number of clinical trials evaluating the prognostic value of effector immune cells for their potent anti‐tumor activity has made such cells the centerpiece of highly effective immunotherapeutic strategies for patients with cancer (Raskov et al., 2021; Stamova et al., 2012). However, the ability of immune checkpoint inhibitors to modulate the function of effector immune cells may vary according to age, and it has been suggested that patients older than 75 years may benefit less than younger patients (Wong et al., 2021). This may be related to so‐called immunosenescence, a phenomenon that refers to reduced immunity with older age.

More recent data has confirmed that effector immune heterogeneity is derived from a diverse array of sources, including demographic factors such as age and sex, epigenetic changes, environmental factors, and selective pressures (Jia et al. 2022). Such features present a significant treatment challenge for a “one‐size‐fits‐all” approach as the unique biology exhibited by an individual's effector immune response can considerably influence the success of the given immunotherapy. Additionally, we found that mTORC1 overexpression in T cells is seen specifically in older women, a demographic with a higher incidence of autoimmune disease (Angum et al., 2020; Kronzer et al., 2021). Given the efficacy of mTOR inhibitors in treatment of autoimmune diseases (Geier & Perl, 2021) such as systemic lupus erythematosus, this finding provides a meaningful clinical‐biologic correlate that could be further leveraged to guide clinical management and drug development. Our results build on multiple hypotheses by exposing the contours of (effector) immune gene expression across aging and sex, highlighting the need for personalized therapeutic strategies that recognize and integrate the heterogeneity of individual patients' steady‐state effector immune responses.

After freezing of our discovery atlas for analysis, another large study of aging in approximately 2 million peripheral blood immune cells from 166 healthy donors appeared (Terekhova et al., 2023). While largely concordant with respect to inference of homeostatic remodeling of the immune system with age, primarily engaging effector lymphocytes, quantitative comparison of our results with their study is difficult. They opted for an aggressive approach to cell cluster annotation, identifying 55 cell types or states, almost three‐times the number we consider. Defining cell states with respect to key marker genes, several of which change with age, shifts the focus away from transcriptional diversity within cell types to changes in cell state abundance. If these offset, then they might obscure the patterns we document in the broader T, B, and myeloid cell types: what one approach regards as changes in abundance of cell states, the other may characterize as elevated heterogeneity with a cell‐type. As with all single cell genomic analyses, inference is heavily influenced by analytical strategy, and there is value in being able to compare orthogonal strategies. Nevertheless, strengths of our approach include the very high replication of the findings across two completely independent atlases, the comprehensive contrast of sex biases, and our evaluation of two hypotheses regarding the source of age‐dependent loss of cell markers.

A limitation of this study is that the data were obtained from cross‐sectional cohorts rather than from longitudinal sampling of the same individuals. As a result, the inference of age differences is based on variability within age classes and does not directly measure changes within individuals over time. Since most of our results pertaining to loss or gain of marker genes are similar in males and females, the study does not yet address reasons for sex‐biased immune aging: it would for example be interesting to evaluate whether XIST‐protein complexes recently implicated in enhanced female autoimmunity (Dou et al., 2024) change in abundance with age, though we did not observe an age difference in XIST transcript levels. Expanded repertiore sequencing may also confirm loss of intra‐individual diversity with age, likely driven by clonal hematopoiesis (Mitchell et al., 2022). Our study also lacks information on the health status of individuals, preventing an analysis of the association between frailty, and immune heterogeneity.

While our findings offer insights into the loss of immune cell identity with age, they remain hypothesis generating and require further functional validation. A decrease in DEGs may suggest a loss of cell identity, but this does not constitute definitive proof. Functional tests are necessary to determine if aged cells with reduced DEGs function differently from younger cells. Additional experiments are crucial to validate our findings and confirm that observed changes in gene expression and cell proportions lead to functional impairments. These further investigations will be essential to substantiate our conclusions and enhance our understanding of immune aging.

Neverthless, by generating atlases of collectively approaching 4 million cells from over 150 individuals per age decile, we are able to place results from focused human studies and model organisms in the larger context of human lifespan. Over the period of several years, individual bulk RNAseq and methylation profiles are remarkably constant (Tabassum et al., 2015), but methylation changes sufficiently with age such that it can be used to estimate chronological age (Horvath & Raj, 2018). We show here that whole blood transcriptomes are generally constant but do undergo a transition starting in the late 30s from naïve T‐cell to cytotoxic T‐cells with accompanying loss of stemness, particularly in females. This shift is due both to changes in cell proportions and gene expression, both favoring greater effector function and antigen presentation with age, but with the proviso that there appears to be a generalized loss of cellular identity, potentially disrupting the network of cellular interactions that support healthy immune function.

## 4 METHODS

### 4.1 Human scRNA‐seq analysis

#### 4.1.1 Single cell PBMC data sources

Our discovery human immune atlas was composed of ~2.7 million PBMCs (peripheral blood mononuclear cells) from 679 individuals sampled in 36 datasets collected from public repositories as listed in Tables S1 and S4, with data downloaded directly from the Gene Expression Omnibus (GEO; https://www.ncbi.nlm.nih.gov/geo), European Nucleotide Archive (ENA; https://www.ebi.ac.uk/ena), or Single Cell Portal (SCP; https://singlecell.broadinstitute.org/single_cell). The selected studies were published between April 2018 and November 2022 and the incorporated datasets were mostly generated on the commonly employed 10× Chromium platform (Zheng et al., 2017: 10× Genomics, 34 studies), although one study used Seq‐well technology (Gierahn et al., 2017). In our selection process, we prioritized studies that employed similar protocols for processing samples and generating data, such as sequencing of PBMCs. From studies contrasting healthy versus diseased, we exclusively included the healthy control samples of the published data. We did not exclude studies that applied flow cytometry‐based cell‐sorting prior to sequencing. The integrated discovery PBMC atlas was generated and analyzed using the following steps: (1) separate pre‐processing and filtering of individual scRNA‐seq datasets from healthy human blood; (2) integration and clustering of PBMCs from each dataset to generate a single healthy human PBMC atlas; (3) validation of annotation of cell clusters; and (4) differential expression analysis. These four steps are described in detail in the following sections, after which we briefly describe the FastDE workflow.

#### 4.1.2 Pre‐processing and filtering of individual scRNA‐seq datasets

A total of 35 scRNA‐seq datasets representing 679 healthy subjects from multiple ancestries, and preprocessed as CellRanger files (Zheng et al., 2017) were analyzed individually incorporating metadata listed in Table S4. To ensure comparability for every individual dataset, we only retained genes found in the Ensembl human (GRCh38) gene model, and implemented the same basic Seurat single‐cell analysis pipeline (version 4.1.1: Hao et al., 2021) in R (version 4.2.1: Ihaka & Gentleman, 1996). Specifically, for each dataset low quality cells with a high percentage of mitochondrial gene counts (> ~ 5%–20%, depending on proportion of outlier removal for each dataset) and with <500 measured genes were excluded. To mitigate potential doublet inclusion, cells with UMI count above 40,000 and detected genes above 5000 were removed. We performed additional analyses using DoubletFinder (McGinnis et al., 2019) into our pipeline to identify and remove potential doublets from our dataset. DoubletFinder identifies doublets in single‐cell RNA‐seq data by generating artificial doublets from the existing dataset, then using principal component analysis (PCA) to compare the artificial and observed cells, and finally applying a k‐nearest neighbors algorithm to assign doublet scores and identify likely doublet cells. The cell‐ droplets identified as doublets by both outlier cell identification and DoubletFinder were removed from further analysis. This led to a working dataset of 2,742,544 single cells. In addition, the OneK1K atlas (Yazar et al., 2022) was imported without modification as a validation set, for which metadata is provided in Table S5.

#### 4.1.3 Dataset integration for the healthy human PBMC discovery atlas

Next, the individual Seurat objects were processed to identify the 19 major cell clusters identified in the Azimuth routine of Seurat v4 (Hao et al., 2021), and then merged one‐by‐one into a single healthy‐state human PBMC object. After filtering, data in each cell was log normalized using Seurat's “NormalizeData” function (method = “LogNormalize”, scale. factor = 10,000), the 2000 most variable genes were identified, and the “ScaleData” function was used to scale and center the gene expression matrix after regressing out the heterogeneity associated with cell cycle and mitochondrial contamination. For each dataset, the number of principal components used for neighborhood graph construction and dimensional reduction was set at 20. Cell clusters marked by the canonical marker genes for platelets (PPBP) and erythrocytes (HBB) were discarded. All individual datasets devoid of platelets and erythrocytes were then used for integration to create our core discovery PBMC atlas comprising data from 35 studies. Batch effect correction was performed on these processed, merged objects using Seurat's reciprocal PCA (“RPCA”: Stuart et al., 2019) with study ID as the batch term with all other parameters as per default. After integration, Uniform Manifold Approximation and Projection (UMAP: Becht et al., 2019) visualization indicated cells from different studies were well mixed into the shared space (Figure 1d; Figure S8 for OneK1K validation).

#### 4.1.4 Annotation of cell clusters

To simplify the analysis, prior annotations were used as a reference to annotate each cell in each dataset. If prior annotations were not available, cellular identity was determined by finding DE genes for each cluster by the “FastFindAllMarkers” function of the FastDE package (test.use = “wilcox”, min.pct = 0.1, logfc.threshold = 0.5). We applied two complementary methods to confirm the cluster annotations shown in Figure 1C. We first compared the top ranking DE genes of query clusters to the well‐characterized cell type‐specific genes from previous datasets, listed in Table S6. We then assessed cluster annotation by applying the R package Azimuth (Hao et al., 2021) which compares the transcriptome of each single cell to reference PBMC transcriptomic datasets to validate cellular identity. Density plots created using the R package Nebulosa (Alquicira‐Hernandez & Powell, 2021) in Figure 1e illustrate some key clusters. For the OneK1K dataset (Yazar et al., 2022) we accepted the Azimuth annotations of predicted cell type identities as published on the CZI (http://cellxgene.cziscience.com) website from which it was downloaded.

#### 4.1.5 Fast differential expression (DE) analysis for clusters and groups

A crucial decision with respect to DE analysis is whether to preserve the intrinsic makeup of cells and samples within the dataset or to use a more structured design in which the numbers of cells and individuals are down‐sampled and balanced prior to performing the contrast of interest. Recent work has established that a significant correlation between the number of DE genes and the number of cells per cell type in both simulated and experimental datasets (Skinnider et al., 2021; Murphy et al., 2023). This relationship was highlighted in findings by Skinnider et al. (2021) in Figures 1e and Extended Data Figure 1j of their publication. To reduce the influence of uneven cell type proportions, we chose to divide samples into deciles based on cell number and subject number based on pilot analyses reported in Figure S1 exploring the impacts of imbalanced sex ratio, sample number, and cell number. The results show that the total number of sex‐specific DEGs (in memory B cells) varies according to the number of cells (test 3); and the ratio of male to female samples (test 1), but not the total number of cells (test 2). In our analyses, differences in the number of cells (test 3: 5K, 12K, or 25K) showed substantial effects on the total number of sex‐specific DEGs, with smaller number of cells tending to show higher DEG numbers. On the same scale, the balance of samples (test 1: 25 females:75 males or 50 females:50 males) showed a similar magnitude of effect on DEGs detected, with nearly a 2‐fold increase in the total number of DEGs when samples were unbalanced as compared to balanced even after adjusting for cell number. Analysis of the total number of samples (test 2: 100 or 200 males and females) exhibited little change in the total number of DEGs, indicating that there is no tendency for genes that are DE to be subject to sample size differences.

Thus, to account for any potential effect of variable group‐specific cell numbers on gene expression, cells were randomly down‐sampled across age and sex subgroups so that the total cell‐type count was the same across each sex decile. From the parent Seurat objects of both the Discovery and the OneK1K atlases, healthy subjects were divided into 20 groups (10 age deciles for males and 10 age deciles for females) according to sex and the age distributions for each cohort (Figure 2c,d). Note that the differences in decile ranges mostly reflect the differences in the age pyramids of the Discovery and OneK1K cohorts, shown in Figure 2a,b. Variability of cell type proportions among contributing studies to the discovery atlas and batches of the OneK1K cohort are shown in Figures S10 and S11 respectively.

Differential expression analysis for each cell type versus all other cells within a sex decile was performed using the Wilcoxon‐test as implemented in the “FastFindAllMarkers” function of the FastDE package. DEGs were identified using the following criteria: (1) a logfold change >0.50, (2) adjusted *p* < 0.05, (3) transcripts detected in >10% of cells in either test group. Plots for the DE genes were generated by customized R code using ggplot2 (v3.3.3, R package).

### 4.2 FastDE design and implementation

#### 4.2.1 Rationale for developing FastDE

The most widely used open‐source software for single cell transcriptome profiling in R is Seurat (Hao et al., 2021; Stuart et al., 2019). The typical workflow is to read in data, scale and transform, perform principal and canonical correlation analysis to identify cell clusters, project these onto 2‐dimensional space with UMAP, and then compute Fold Change and Wilcoxon Rank Sum Tests to find cluster‐specific marker genes. Seurat defaults to LIMMA (Ritchie et al., 2015) for small datasets, but uses native R code for larger ones, and converts sparse to dense matrices, which places constraints on computational time and the size of dataset that can be analyzed. Incremental versions of the software have improved clustering performance and incorporated options for differential expression analysis, but the FindMarkers function remains the rate limiting step for analysis.

We ran Seurat on a single core of a Xeon E7‐8870 processor with public datasets containing between 3000 and 68,000 immune cells of varying complexity, and consistently observed that between 79% and 97% of the run‐time was consumed by the FindMarkers function (Table S8). The R language (Ihaka & Gentleman, 1996) is limited to 2 billion non‐zero elements in a sparse matrix and is therefore constrained for analyzing datasets with high cell counts. In addition, we experienced intermittent memory allocation failures when using multiple cores, and analysis of a dataset of 600,000 cells failed to identify DE genes. To address these limitations, we recoded the key components of Seurat's FindMarkers implementation in C++ (Stroustrup, 2013) utilizing sparse matrix specific algorithms, large sparse matrix indices, and OpenMP parallelization (Chandra et al., 2001) to accelerate the workflow, thereby facilitating analysis of very large compendium datasets and improving multicore processing speed and stability.

#### 4.2.2 Overview of the FastDE workflow

FastDE is implemented as an R package with C++ for compute intensive components. The C++ components include Wilcoxon Rank Sum test, find markers, fold change, and supporting functions such as sparse matrix transpose and sums. The R FastDE package provides R language binding for the C++ functions as well as high level functions meant as direct replacement of the Seurat “FoldChange” and “FindMarkers” functions. FastDE's core C++ implementation is available from https://github.com/tcpan/fastde‐cpp. The FastDE R package utilizes the C++ implementation and is available as source from https://github.com/tcpan/fastde and will be made available through the CRAN R‐repository.

The Wilcoxon Rank Sum test algorithm is illustrated in Figure S9. Full documentation is provided with the github repository. It computes for each gene the rank sums and *p*‐values of every class label in a one‐versus‐all‐others fashion. The gene expression counts are stored in a sparse matrix, with each column corresponding to a gene. For each column, we sort the non‐zero expression values, assign ranks, then sum the ranks by class using a hash table. Where there exist ties in the sorted array, the algorithm uses the median of the ranks, and computes the tie corrections. Once the rank sums and tie corrections have been computed, the U statistics for Wilcoxon Rank Sum test are readily computed for each class, which are then converted to *p*‐values via *z*‐transform and C language built‐in Gaussian complementary error function. The algorithm returns a *p*‐value matrix, where each row corresponds to a class label and each column a gene. A *p*‐value in the matrix indicates whether the expression counts of the cells with a particular class label is statistically different than those of other cells for a particular gene, and the existing Seurat logic can be used to identify the DE genes for each class. Our implementation is fully parallelized for multi‐core, multithread environments.

Seurat's “FindAllMarkers” implementation operates on a gene column for one class at a time, using a nested loop that iterates over the cell classes in the outer loop, and processing the gene columns in the inner loop using the “FoldChange” and “FindMarkers” functions. In this configuration, the sorted column therefore cannot be reused for multiple classes. FastDE's implementation of “FindMarkers” and “FoldChange” incorporates the entire nested loops and inverts the nesting order, iterating over the gene columns in the outer loop, and computes the rank sums and *p*‐values for all cell classes simultaneously in the inner loop.

#### 4.2.3 Evaluation and validation of FastDE

The performance evaluation was conducted using a system with four Intel(R) Xeon(R) E7‐8870 v3 CPUs each with 18‐cores for a total of 72 CPU cores and 1 TB of DDR4 ECC memory. The operating system used was Ubuntu 18.04.6 LTS. A 224GB NVME SSD was used to host the datasets and the pipeline outputs.

R version 4.2.2 and Seurat 4.3.0 were used in the Seurat and FastDE evaluation. R packages required for Seurat and FastDE were installed through the R Bioconductor and devtools packages. FastDE's FoldChange, FindMarkers, and Wilcoxon Rank Sum Test implementations were validated using randomly generated sparse matrices. The outputs of the Wilcoxon Rank Sum test were compared to the corresponding R functions, and the means, standard deviation, and range of the value differences, as well as root mean square error were computed and shown to be within R's default tolerance of approximately 1.5×10^−8^. FastDE and Seurat's “FoldChange” functions show higher variability due to differences in R and C++'s rounding policy for negative numbers.

PBMC datasets were chosen with progressively higher cell counts for assessing the scalability of the computational performance of FastDE and Seurat. Six datasets with approximately 3 K, 6 K, 8 K, 10 K, 33 K, 68 K, and 600 K cells were included in the evaluation. Details of the datasets will be provided upon request. A typical Seurat single cell analysis pipeline (https://satijalab.org/seurat/articles/pbmc3k_tutorial.html) was used for the evaluations. The pipeline steps include data set loading, dimensionality reduction (RunPCA), neighbor finding (FindNeighbors) and clustering, visualization (RunUMAP), and differential gene expression analysis. Default parameters were used for all the steps, including algorithm choices: PCA for dimensionality reduction, Louvain for clustering, and UMAP for visualization, except that ScaleData used only variable genes and 30 principal components instead of 50 were included for RunPCA, FindNeighbors, and RunUMAP. Run times for the pipelines, excluding file I/O times, as well as the differential gene expression analysis step were captured using the tictoc package in R. Each run was on 1 or 64 cores, and repeated three times and the average reported in Table S8. The table shows the scalability and performance of FastDE compared to Seurat.

### 4.3 Evaluation of cell type dispersion

To calculate cell–cell heterogeneity as a measure of intra‐cell type dispersion, cells in each cell type were split into subsets based on their sex and age‐group origin (<40 years old or >79 years old). We used the R package disscat (https://rdrr.io/github/sbrn3/disscat/: Anonymous, 2019) to identify the centroid within each cell‐type for each age and sex group subset; the Euclidean distance from each cell to the centroid within the subset was calculated based on PCA gene expression space. For the cell type convergence analysis, the Euclidean distance from each cell to the centroid across all cell types was computed on the PCA gene expression space (dimensions 1:10) for each cell‐type and age/sex group.

### 4.4 Aging score analysis

To further assess the impact of aging on circulating T cells, we selected the top 30 genes of the 116 total genes that were DE and shared between sexes in the T cell compartment. We calculated aging scores for every cell by averaging the scaled (Z‐normalized) expression levels of genes in our predefined list. To compute these scores, we summed the unique molecular identifiers (UMI) for all aging genes expressed within a given cell subset (denoted as X) and divided this sum by the total UMI expressed by cells in subset X (Andreatta & Carmona, 2021).

### 4.5 Gene functional annotation

Gene ontology and KEGG pathway analyses of DEGs were performed using the R package topGO (v.2.38.1). GO biological process terms with Bonferroni‐corrected *p* values (FDR) <0.05 were considered as significantly enriched terms.

## AUTHOR CONTRIBUTIONS

EC conceived and assembled the atlas, performed the bioinformatic analysis, and helped write the manuscript. TP conceived and programmed FastDE, with assistance from MA and SC. VD provided critical feedback on the analyses and writing. GG drafted the manuscript and helped coordinate the project.

## FUNDING INFORMATION

This research was supported in part by an IRAD Pilot Grant from the Georgia Tech Research Institute (TP, MA) and training grant 1T32EB021962‐01A1 (EC, Julia Babensee PI).

## CONFLICT OF INTEREST STATEMENT

The authors declare that they have no conflicts of interest.

## Supporting information


Data S1.

Table S1.

Table S2.

Table S3.

Table S4.

Table S5.

Table S6.

Table S7.

Table S8.

## Data Availability

All data analyzed in this manuscript are taken from open access datasets as documented in the Tables S1 and S4 for the Discovery atlas, or from the website of the CZI (http://cellxgene.cziscience.com) for the OneK1K study. Access to the Seurat objects for this study will be provided upon request to the authors.
