# Supplementary material for: Loss of immune cell identity with age inferred from large atlases of single cell transcriptomes
Source: Aging Cell. 2024 Aug 14;23(12):e14306. doi: 10.1111/acel.14306 (PMC11634704; doi:10.1111/acel.14306)
Supplement: Supplementary file 1 — Data S1. [file ACEL-23-e14306-s005.pdf]

LIST OF SUPPLEMENTAL FIGURES

Figure S1. Age class imbalance effect on FindMarkers

Figure S2. Concordance of cell type annotation after integration in the discovery atlas

Figure S3. Changes in cellular proportions with age in the discovery atlas

Figure S4. Changes in cellular identity with age in the discovery atlas

Figure S5. Replication of changes in cellular identity with age in the OneK1K atlas

Figure S6. Variance partitioning of pseudobulk profiles in the OneK1K atlas

Figure S7. Changes in cellular identity with age per individual

Figure S8. UMAP visualization of OneK1K atlas

Figure S9. FastDE Workflow

Figure S10. Proportion of each cell type in each pool number in the OneK1K atlas

Figure S11. Proportion of each cell type in each study in the discovery atlas

LIST OF SUPPLEMENTAL TABLES

Table S1. List of studies included in the discovery atlas

Table S2. Complete list of cell-type specific markers identified by FastFindAllMarkers

Table S3. List of differentially expressed genes in OneK1K atlas comparisons

Table S4. Metadata for the discovery atlas

Table S5. Metadata for the OneK1K atlas

Table S6. List of classical PBMC cell type markers used for annotation

Table S7. Cell-type specific markers identified by FastFindAllMarkers for each age and sex subgroup

Table S8. Summary statistics for FastDE workflow

SUPPLEMENTAL FIGURES

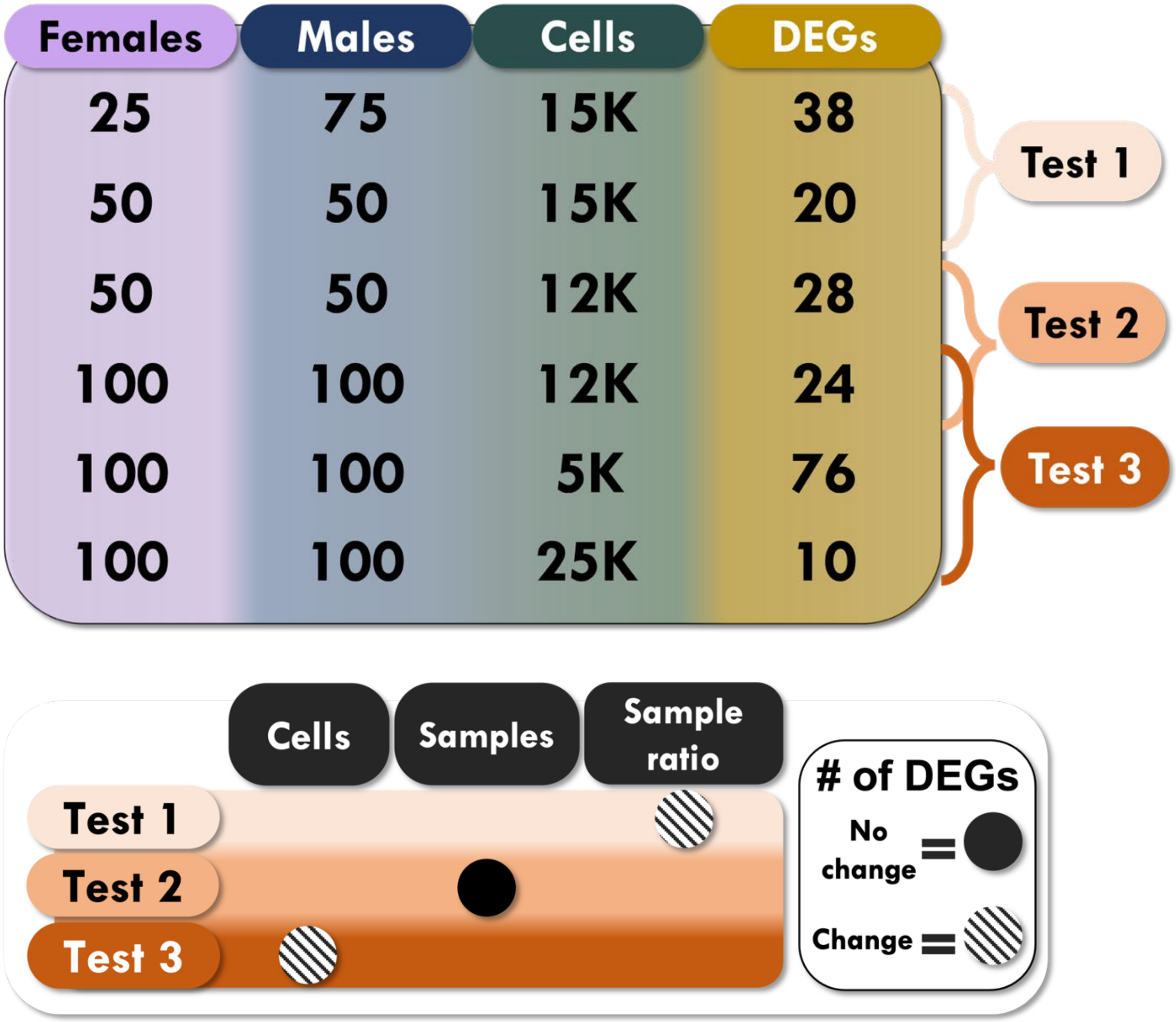

Figure S1. Justification for dividing samples by decile rather than age group. Sex-specific DEGs in memory B cells were calculated according to the balance of samples (test 1: 25 females:75 males or 50 females:50 males); total number of samples (test 2: 100 or 200 males and females); and the number of cells (test 3: 25K or 5K cells). Bottom legend summarizes the effect of each test on the total number of sex-specific DEGs.

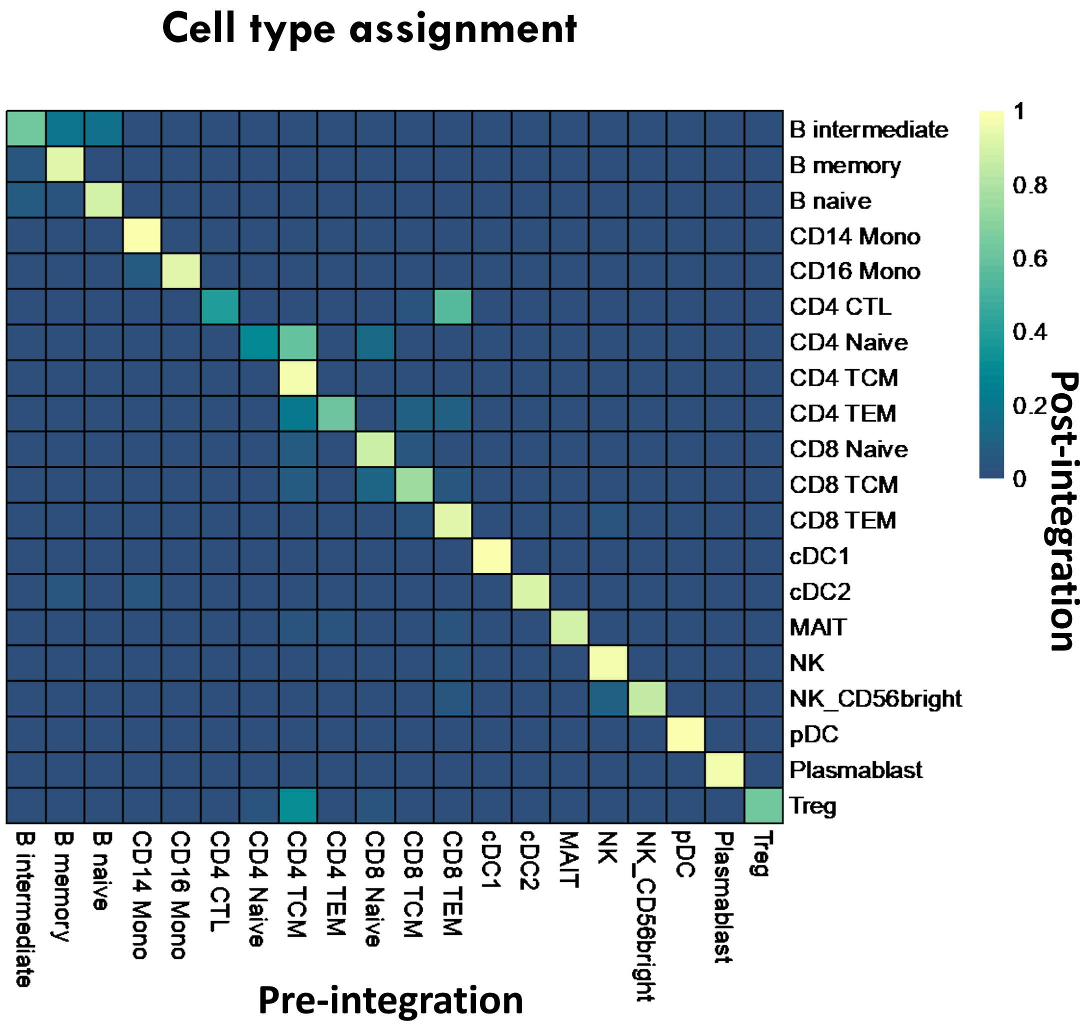

Figure S2 Cell-type assignments pre and post integration.

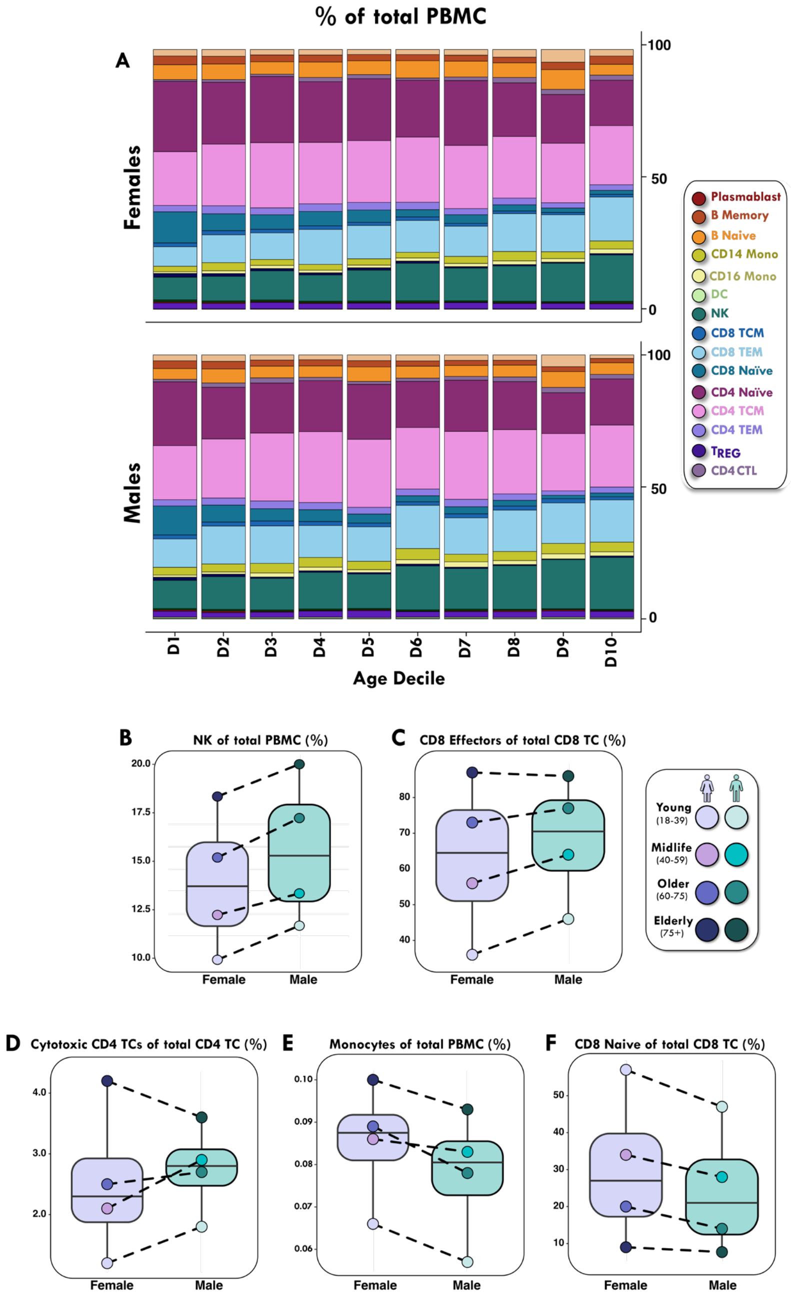

Figure S3. Changes in cell proportions associated with aging and sex. (a) Relative cell type abundance in males and females across the ten age deciles. (b) Percentage of NK in PBMCs across age and sex. Age groups includes young (18-39 years), middle age (40-59 years), older (60-75 years), elderly (75+ years). (c) Percentage of CD8 effectors of total CD8 TC across age and sex. (d) Percentage of CD4 TCs of total CD4 TC across age and sex. (e) Percentage of Monocytes in PBMCs across age and sex. (f) Percentage of CD8 naive of total CD8 TC across age and sex.

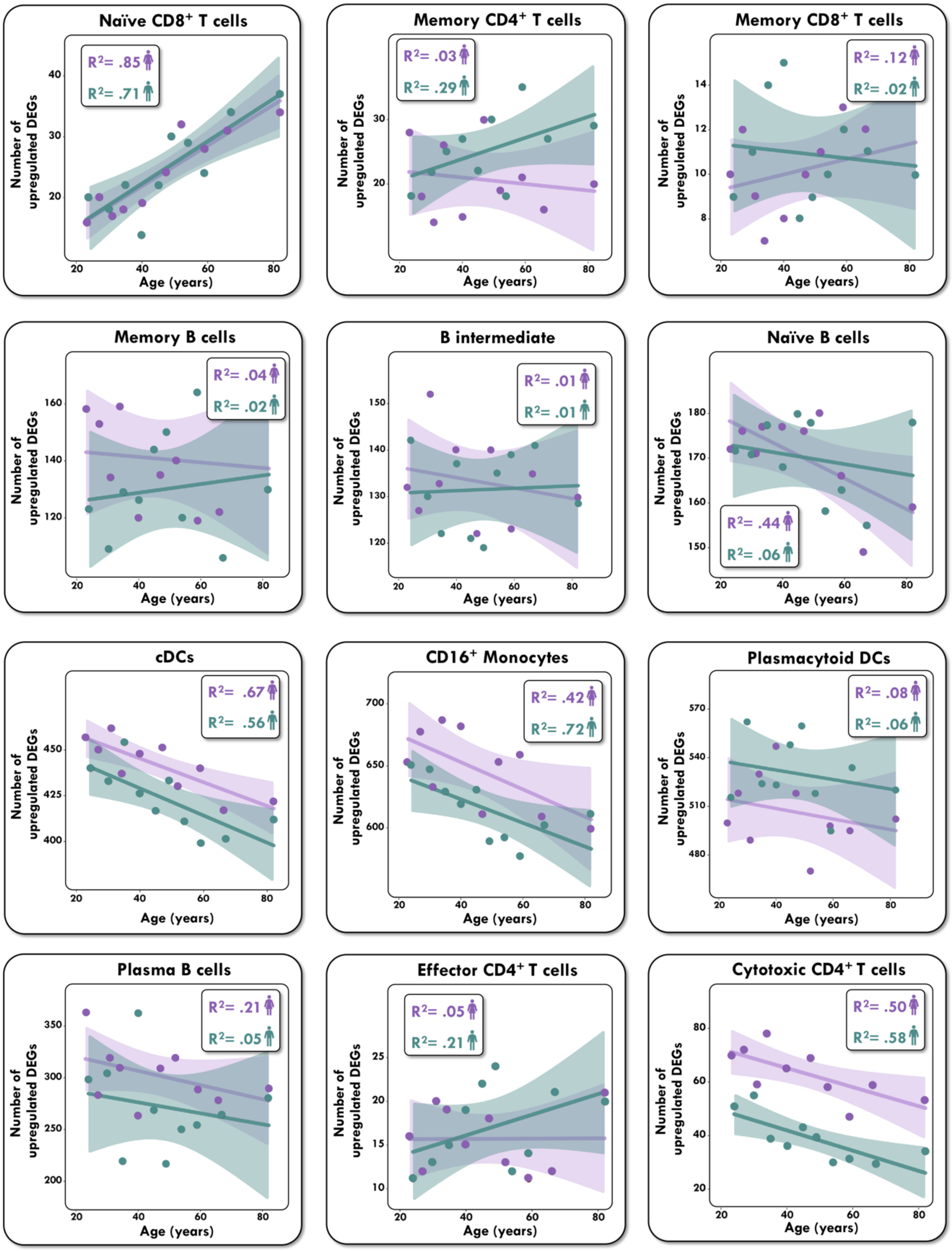

Figure S4. Extended association of cell type identity with age in the discovery atlas. No correlation between age and the number of cell-type specific markers in 12 immune cell types.

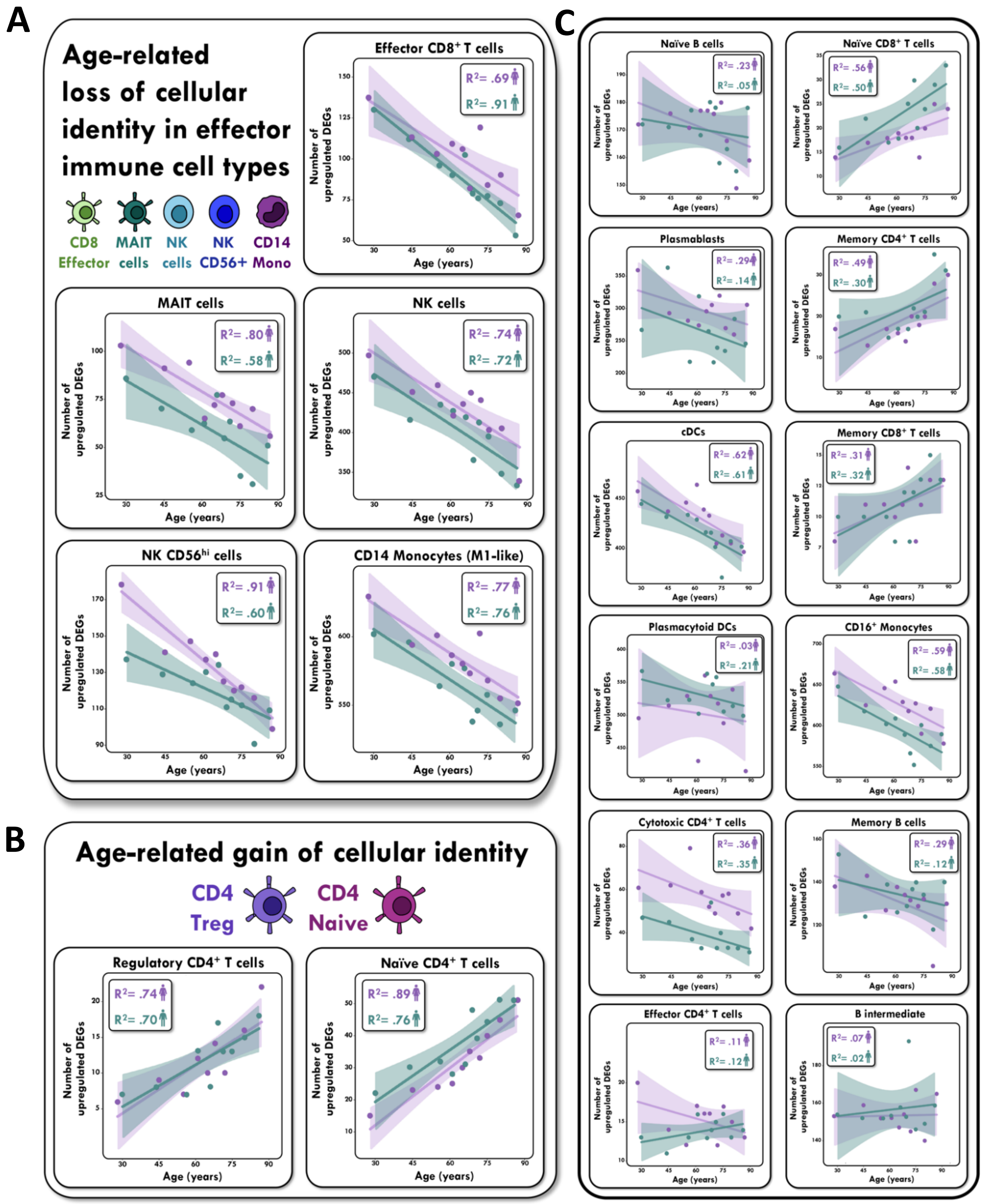

Figure S5. Replication cell type identity associations with age in the OneK1K atlas. (A) Positive correlation between age and the number of cell-type specific markers in effector immune cell types. (B) Negative correlation between age and the number of cell-type specific markers in CD4 regulatory T cells and CD4 Naïve T cell. (C) No correlation between age and the number of cell-type specific markers in the other 12 immune cell types.

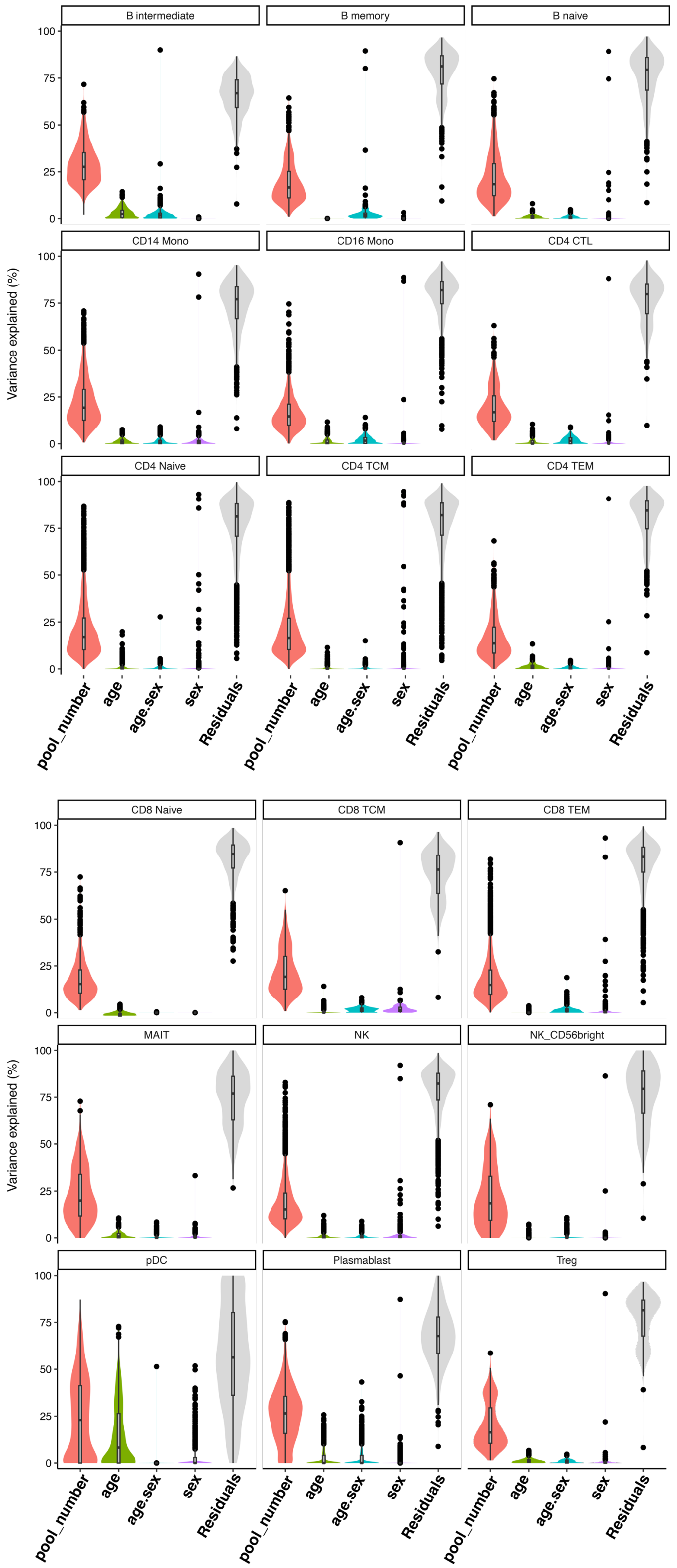

Figure S6. Variance partitioning analysis of the OneK1K. Violin plots summarizing variance partitioning analysis separating the fraction of expression variation for each gene into 5 components.

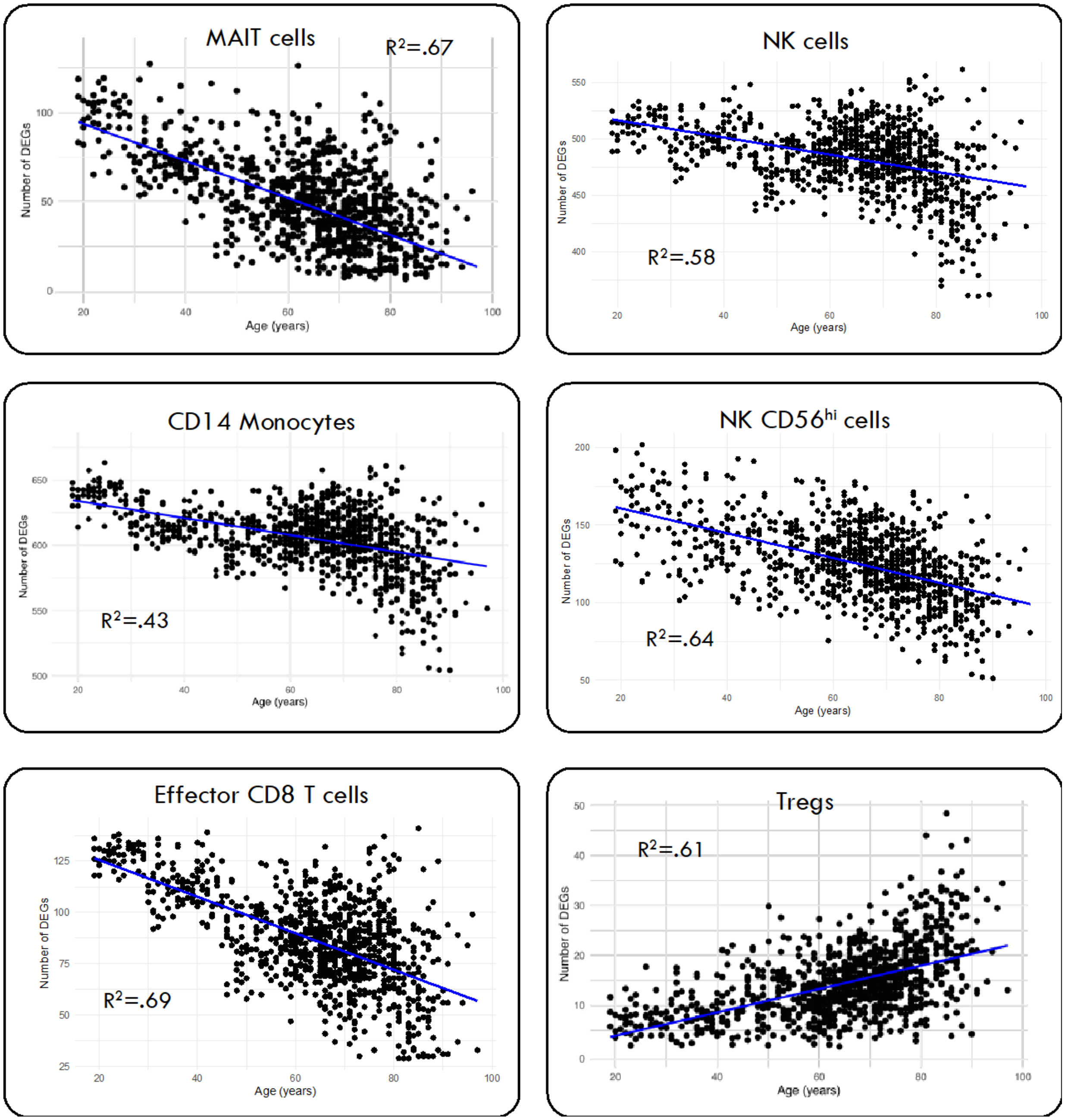

Figure S7. Changes in cellular identity with age per individual. The regression plot shows the number of differentially expressed genes (DEGs) per cell type versus age for each individual, calculated using Wilcoxon Rank Sum methods on each donor's single cell profile deciles, highlighting the variation in DEG counts among individuals within each age group.

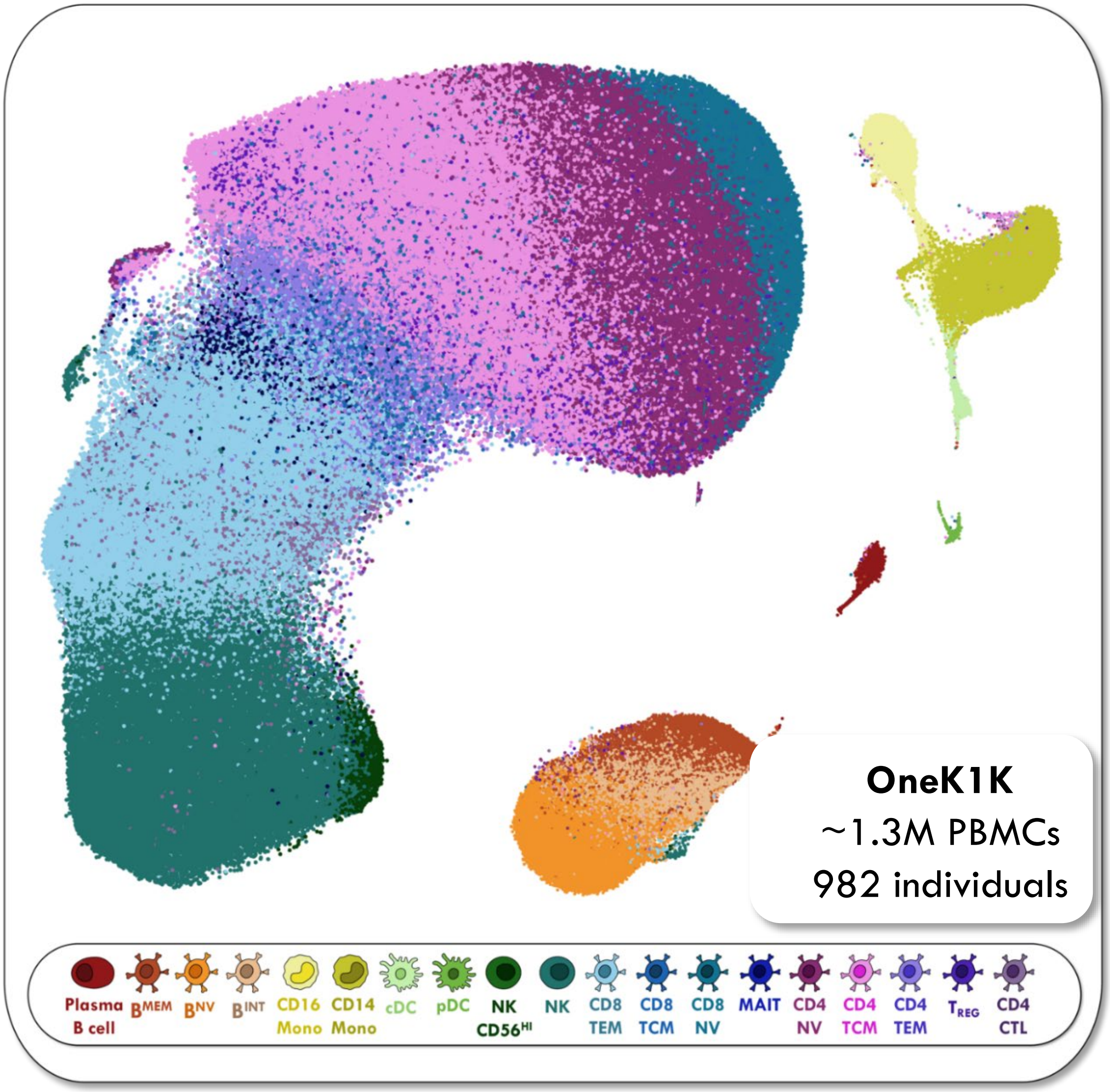

Figure S8. UMAP visualization of OneK1K Atlas (Yazar et al 2022). UMAP plot showing 1,267,758 PBMCs plotted from all 982 individuals, with clustering of the 19 cell types shown in the bottom legend.

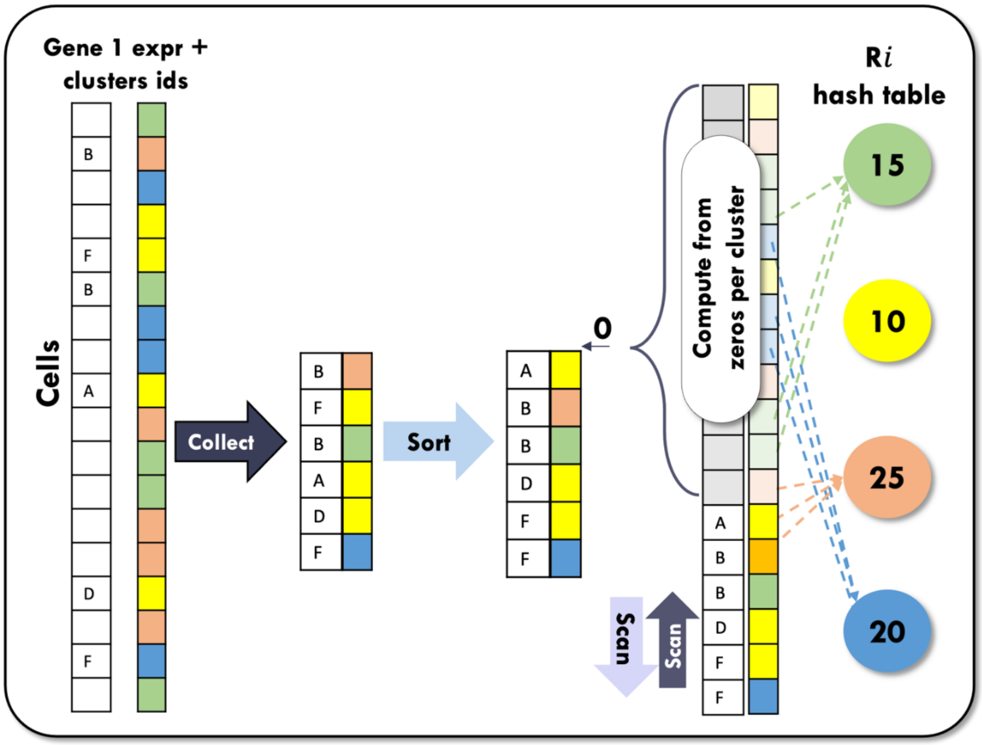

Figure S9. FastDE algorithm for computing the rank sums in the Wilcoxon Rank Sum test.

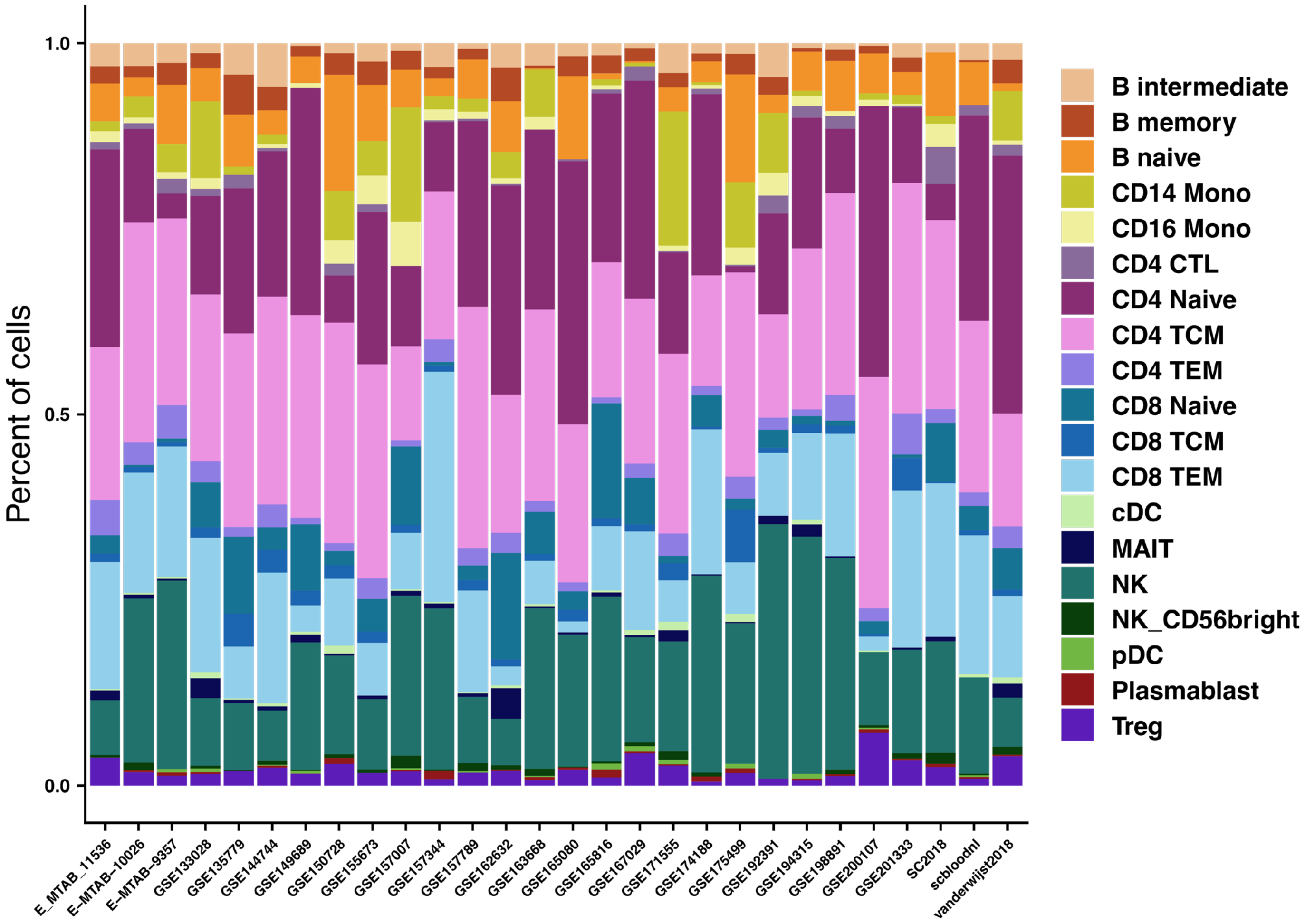

Figure S10. Proportion of each cell type in each study in the discovery atlas

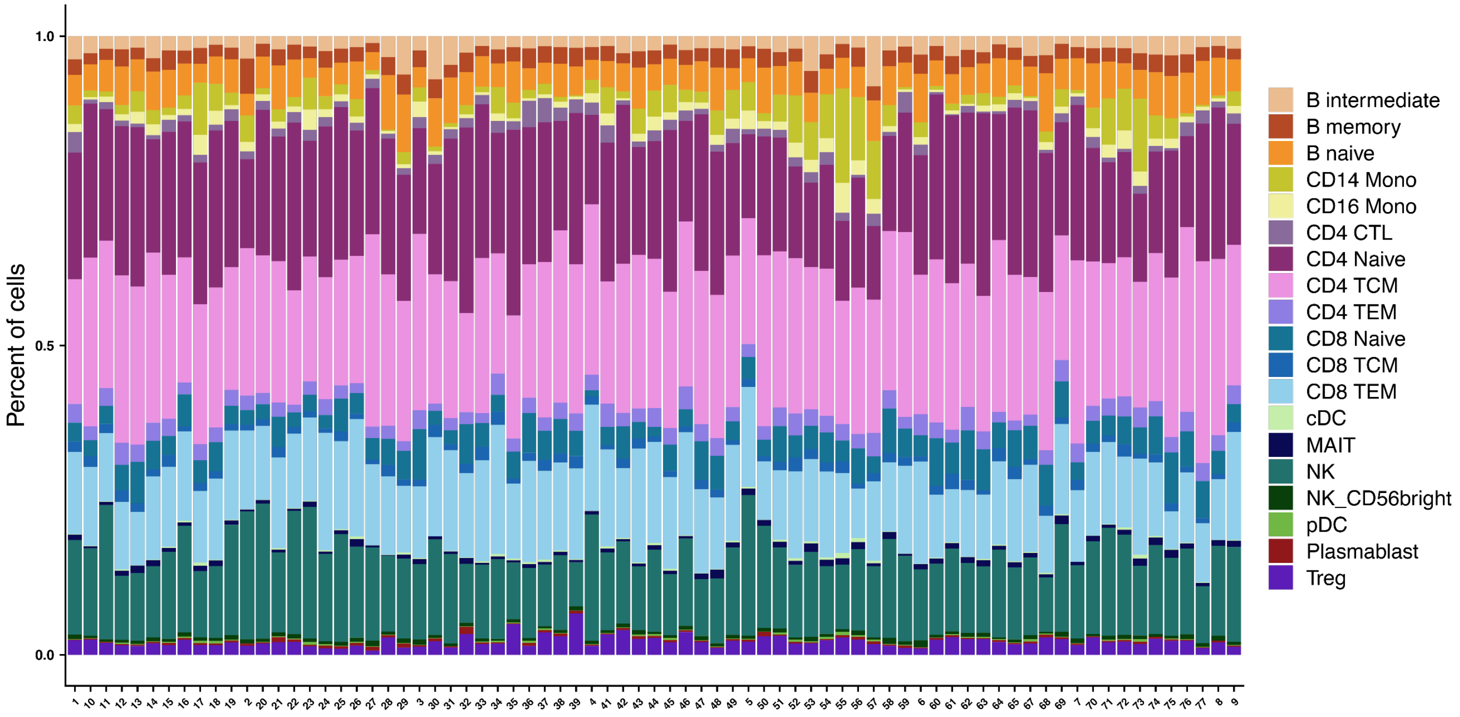

Figure S11. Proportion of each cell type in each pool number in the OneK1K atlas

SUPPLEMENTAL TABLES

Table S6. Classical Markers Used for Cell Type Identification

Cell type	Markers
B intermediate	MS4A1, TNFRSF13B, IGHM, IGHD, AIM2, CD79A, LINC01857, RALGPS2, BANK1, CD79B
B memory	MS4A1, COCH, AIM2, BANK1, SSPN, CD79A, TEX9, RALGPS2, TNFRSF13C, LINC01781
B naive	IGHM, IGHD, CD79A, IL4R, MS4A1, CXCR4, BTG1, TCL1A, CD79B, YBX3
Plasmablast	IGHA2, MZB1, TNFRSF17, DERL3, TXNDC5, TNFRSF13B, POU2AF1, CPNE5, HRASLS2, NT5DC2
CD4 CTL	GZMH, CD4, FGFBP2, ITGB1, GZMA, CST7, GNLY, B2M, IL32, NKG7
CD4 Naive	TCF7, CD4, CCR7, IL7R, FHIT, LEF1, MAL, NOSIP, LDHB, PIK3IP1
CD4 TCM	IL7R, TMSB10, CD4, ITGB1, LTB, TRAC, AQP3, LDHB, IL32, MAL
CD4 TEM	IL7R, CCL5, FYB1, GZMK, IL32, GZMA, KLRB1, TRAC, LTB, AQP3
Treg	RTKN2, FOXP3, AC133644.2, CD4, IL2RA, TIGIT, CTLA4, FCRL3, LAIR2, IKZF2
CD8 Naive	CD8B, S100B, CCR7, RGS10, NOSIP, LINC02446, LEF1, CRTAM, CD8A, OXNAD1
CD8 TCM	CD8B, ANXA1, CD8A, KRT1, LINC02446, YBX3, IL7R, TRAC, NELL2, LDHB
CD8 TEM	CCL5, GZMH, CD8A, TRAC, KLRD1, NKG7, GZMK, CST7, CD8B, TRGC2
cDC	FCER1A, CST3, SERPINF1, HLA-DQA1, CLEC10A, CD1C, ENHO, PLD4, GSN, SLC38A1, NDRG2, AFF3
pDC	ITM2C, PLD4, SERPINF1, LILRA4, IL3RA, TPM2, MZB1, SPIB, IRF4, SMPD3
CD14 Mono	S100A9, CTSS, S100A8, LYZ, VCAN, S100A12, IL1B, CD14, G0S2, FCN1
CD16 Mono	CDKN1C, FCGR3A, PTPRC, LST1, IER5, MS4A7, RHOC, IFITM3, AIF1, HES4
NK	GNLY, TYROBP, NKG7, FCER1G, GZMB, TRDC, PRF1, FGFBP2, SPON2, KLRF1
NK_CD56bright	XCL2, FCER1G, SPINK2, TRDC, KLRC1, XCL1, SPTSSB, PPP1R9A, NCAM1, TNFRSF11A
MAIT	KLRB1, NKG7, GZMK, IL7R, SLC4A10, GZMA, CXCR6, PRSS35, RBM24, NCR3

Table S8. Summary Statistics for Workflow Acceleration using FastDE**.**

CPU cores	Dataset (k cells)	FastDE FindMarkers (sec)	FastDE Pipeline (sec)	Seurat v4.3 FindMarkers (sec)	Seurat v4.3 Pipeline (sec)	Speed up FindMarkers	Speed up Pipeline
1	3	1.27	13.09	85.19	97.12	67.25	7.42
	6	1.88	23.72	157.54	179.88	83.80	7.58
	8	4.66	33.75	471.36	501.47	101.10	14.86
	10	9.34	39.82	1857.95	1890.12	198.88	47.47
	33	9.44	118.57	1586.77	1697.63	168.14	14.32
	68	13.54	232.67	2617.93	2839.41	193.41	12.20
	600	230.21	2875.51	145565.11	148244.85	632.31	51.55
64	3	1.16	11.70	19.42	30.12	16.68	2.57
	6	1.20	19.41	30.11	48.95	25.13	2.52
	8	1.98	29.56	79.36	108.32	40.04	3.66
	10	3.11	32.57	225.56	257.44	72.46	7.91
	33	2.82	88.39	191.24	279.27	67.87	3.16
	68	3.50	196.80	335.51	532.24	95.79	2.70
	600	28.73	2503.57	2805.33	5328.63	97.63	2.13

The run times are reported in seconds and speed up are calculated as ratios of Seurat's times to FastDE's times. The pipelines were executive using 1 core or 64 cores on 4 Xeon E7-8870 CPUs for the FindMarkers function and the complete pipeline. Pipeline run times exclude file input and output times.
