# Supplementary material for: Loss of immune cell identity with age inferred from large atlases of single cell transcriptomes
Source: Aging Cell. 2024 Aug 14;23(12):e14306. doi: 10.1111/acel.14306 (PMC11634704; doi:10.1111/acel.14306)
Supplement: Supplementary file 7 — Table S6. [file ACEL-23-e14306-s003.docx]

**Supplementary Table 6** **Classical Markers Used for Cell Type Identification**

| **Cell type** | **Markers** |
| --- | --- |
| B intermediate | MS4A1, TNFRSF13B, IGHM, IGHD, AIM2, CD79A, LINC01857, RALGPS2, BANK1, CD79B |
| B memory | MS4A1, COCH, AIM2, BANK1, SSPN, CD79A, TEX9, RALGPS2, TNFRSF13C, LINC01781 |
| B naive | IGHM, IGHD, CD79A, IL4R, MS4A1, CXCR4, BTG1, TCL1A, CD79B, YBX3 |
| Plasmablast | IGHA2, MZB1, TNFRSF17, DERL3, TXNDC5, TNFRSF13B, POU2AF1, CPNE5, HRASLS2, NT5DC2 |
| CD4 CTL | GZMH, CD4, FGFBP2, ITGB1, GZMA, CST7, GNLY, B2M, IL32, NKG7 |
| CD4 Naive | TCF7, CD4, CCR7, IL7R, FHIT, LEF1, MAL, NOSIP, LDHB, PIK3IP1 |
| CD4 TCM | IL7R, TMSB10, CD4, ITGB1, LTB, TRAC, AQP3, LDHB, IL32, MAL |
| CD4 TEM | IL7R, CCL5, FYB1, GZMK, IL32, GZMA, KLRB1, TRAC, LTB, AQP3 |
| Treg | RTKN2, FOXP3, AC133644.2, CD4, IL2RA, TIGIT, CTLA4, FCRL3, LAIR2, IKZF2 |
| CD8 Naive | CD8B, S100B, CCR7, RGS10, NOSIP, LINC02446, LEF1, CRTAM, CD8A, OXNAD1 |
| CD8 TCM | CD8B, ANXA1, CD8A, KRT1, LINC02446, YBX3, IL7R, TRAC, NELL2, LDHB |
| CD8 TEM | CCL5, GZMH, CD8A, TRAC, KLRD1, NKG7, GZMK, CST7, CD8B, TRGC2 |
| cDC | FCER1A, CST3, SERPINF1, HLA-DQA1, CLEC10A, CD1C, ENHO, PLD4, GSN, SLC38A1, NDRG2, AFF3 |
| pDC | ITM2C, PLD4, SERPINF1, LILRA4, IL3RA, TPM2, MZB1, SPIB, IRF4, SMPD3 |
| CD14 Mono | S100A9, CTSS, S100A8, LYZ, VCAN, S100A12, IL1B, CD14, G0S2, FCN1 |
| CD16 Mono | CDKN1C, FCGR3A, PTPRC, LST1, IER5, MS4A7, RHOC, IFITM3, AIF1, HES4 |
| NK | GNLY, TYROBP, NKG7, FCER1G, GZMB, TRDC, PRF1, FGFBP2, SPON2, KLRF1 |
| NK_CD56bright | XCL2, FCER1G, SPINK2, TRDC, KLRC1, XCL1, SPTSSB, PPP1R9A, NCAM1, TNFRSF11A |
| MAIT | KLRB1, NKG7, GZMK, IL7R, SLC4A10, GZMA, CXCR6, PRSS35, RBM24, NCR3 |
