# Supplementary material for: Loss of immune cell identity with age inferred from large atlases of single cell transcriptomes
Source: Aging Cell. 2024 Aug 14;23(12):e14306. doi: 10.1111/acel.14306 (PMC11634704; doi:10.1111/acel.14306)
Supplement: Supplementary file 9 — Table S8. [file ACEL-23-e14306-s001.docx]

**Supplementary Table 8. Summary Statistics for Workflow Acceleration using FastDE**

1. **Single core Processing**

| Dataset (k cells) | FastDE | | Seurat v4.3 | | Speed up | |
| --- | --- | --- | --- | --- | --- | --- |
|  | FindMarkers (sec) | Pipeline (sec) | FindMarkers (sec) | Pipeline (sec) | FindMarkers | Pipeline |
| 3 | 1.56 | 24.80 | 88.37 | 111.46 | 56.57 | 4.49 |
| 6 | 2.08 | 42.82 | 159.04 | 199.86 | 76.41 | 4.67 |
| 8 | 4.90 | 57.67 | 477.02 | 529.69 | 97.29 | 9.19 |
| 10 | 9.42 | 60.15 | 1839.36 | 1890.08 | 195.28 | 31.42 |
| 33 | 9.31 | 177.90 | 1591.56 | 1760.17 | 170.95 | 9.89 |
| 68 | 13.30 | 388.77 | 2608.77 | 2985.35 | 196.20 | 7.68 |
| 600 | 233.58 | 2574.91 | 145565.11 | 148029.67 | 623.19 | 57.49 |

1. **64 Core Processing**

| Dataset (k cells) | FastDE | | Seurat v4.3 | | Speed up | |
| --- | --- | --- | --- | --- | --- | --- |
|  | FindMarkers (sec) | Pipeline (sec) | FindMarkers (sec) | Pipeline (sec) | FindMarkers | Pipeline |
| 3 | 1.16 | 10.73 | 19.42 | 28.99 | 16.68 | 2.70 |
| 6 | 1.20 | 18.52 | 30.11 | 47.43 | 25.13 | 2.56 |
| 8 | 1.98 | 27.18 | 79.36 | 104.56 | 40.04 | 3.85 |
| 10 | 3.11 | 28.05 | 225.56 | 250.49 | 72.46 | 8.93 |
| 33 | 2.82 | 84.03 | 191.24 | 272.45 | 67.87 | 3.24 |
| 68 | 3.50 | 191.02 | 335.51 | 523.03 | 95.79 | 2.74 |
| 600 | 28.73 | 2389.61 | 2805.33 | 5166.20 | 97.63 | 2.16 |

The run times are reported in seconds and speed ups are calculated as ratios of Seurat’s times to FastDE’s times. The pipelines were executive using one core of a Xeon E7-8870 CPU for the FindMarkers function and the complete pipeline. Pipeline run times exclude file input and output times.
